# Phenotypic Variation in the Plant Pathogenic Bacterium *Acidovorax citrulli*

**DOI:** 10.1371/journal.pone.0073189

**Published:** 2013-09-02

**Authors:** Ram Kumar Shrestha, Tally Rosenberg, Daria Makarovsky, Noam Eckshtain-Levi, Einat Zelinger, June Kopelowitz, Johannes Sikorski, Saul Burdman

**Affiliations:** 1 The Department of Plant Pathology and Microbiology and the Minerva Otto Warburg Center for Agricultural Biotechnology, The Robert H. Smith Faculty of Agriculture, Food and Environment, The Hebrew University of Jerusalem, Rehovot, Israel; 2 The Interdepartmental Equipment Facility, The Robert H. Smith Faculty of Agriculture, Food and Environment, The Hebrew University of Jerusalem, Rehovot, Israel; 3 Savyon Diagnostics Ltd., Ashdod, Israel; 4 Leibniz Institut DSMZ-Deutsche Sammlung von Mikroorganismen und Zellkulturen GmbH, Braunschweig, Germany; University of the West of England, United Kingdom

## Abstract

*Acidovorax citrulli* causes bacterial fruit blotch (BFB) of cucurbits, a disease that threatens the cucurbit industry worldwide. Despite the economic importance of BFB, little is known about pathogenicity and fitness strategies of the bacterium. We have observed the phenomenon of phenotypic variation in *A. citrulli*. Here we report the characterization of phenotypic variants (PVs) of two strains, M6 and 7a1, isolated from melon and watermelon, respectively. Phenotypic variation was observed following growth in rich medium, as well as upon isolation of bacteria from inoculated plants or exposure to several stresses, including heat, salt and acidic conditions. When grown on nutrient agar, all PV colonies possessed a translucent appearance, in contrast to parental strain colonies that were opaque. After 72 h, PV colonies were bigger than parental colonies, and had a fuzzy appearance relative to parental strain colonies that are relatively smooth. *A. citrulli* colonies are generally surrounded by haloes detectable by the naked eye. These haloes are formed by type IV pilus (T4P)-mediated twitching motility that occurs at the edge of the colony. No twitching haloes could be detected around colonies of both M6 and 7a1 PVs, and microscopy observations confirmed that indeed the PVs did not perform twitching motility. In agreement with these results, transmission electron microscopy revealed that M6 and 7a1 PVs do not produce T4P under tested conditions. PVs also differed from their parental strain in swimming motility and biofilm formation, and interestingly, all assessed variants were less virulent than their corresponding parental strains in seed transmission assays. Slight alterations could be detected in some DNA fingerprinting profiles of 7a1 variants relative to the parental strain, while no differences at all could be seen among M6 variants and parental strain, suggesting that, at least in the latter, phenotypic variation is mediated by slight genetic and/or epigenetic alterations.

## Introduction

Bacteria have developed mechanisms that allow them to adapt and maintain cell viability in rapidly changing environments. These mechanisms, that are associated with genetic or epigenetic alterations and phenotypic variation, include so-called adaptive mutations and phase variation [1,2]. Adaptive mutations, also called stationary-phase mutations, occur during periods of prolonged, non-lethal stresses [3]. They can occur as result of transposon-mediated insertions and deletions, substitutions, point mutations and gene amplification [4], and have been associated with development of antibiotic resistance and colonization of new hosts [5]. In phase variation, the expression of a given gene is either in an 'ON' or in an 'OFF' mode. These events are often reversible; however, in some cases they were found to be irreversible [6–8]. Phase variation has been defined as a random event that occurs at high frequency (much higher than spontaneous mutations), involves changes in the DNA and leads to a phenotypically heterogeneous population [9,10]. It has been studied extensively in animal pathogenic bacteria in relation to extracellular polysaccharide (EPS) and lipopolysaccharide (LPS) composition, surface proteins, toxins, flagella, colony morphology and more [11].

The molecular mechanisms associated with phase variation are diverse and include genetic, epigenetic and environmental regulation. DNA inversions, duplications and deletions, transpositions, homologous recombination, slipped-strand mispairing and differential methylation can be associated with phase variation [10,12,13]. Phase variation can also occur as result of changes in the expression of global regulatory proteins [11,14,15]. In animal pathogenic bacteria, it has been proposed that phase variation, which is often linked with antigenic variation, increases bacterial fitness, and contributes to niche adaptation or avoidance of host defenses [1].

Modifications in the DNA are involved in both adaptive mutations and phase variation, and there is a controversy in the literature around definition of these phenomena. According to Parsek and Singh [16], phase variation occurs as a result of DNA rearrangements at specific sites in the bacterial genome, while adaptive mutations are mediated by point mutations, recombinations and transposition events at a larger scale. Similarly, phase variation and adaptive mutations have been considered as different phenomena by several other scientists [17,18]. In contrast, others referred to as adaptive mutation as one of the mechanisms that can lead to the development of phase variation in bacterial pathogens [5,19]. Due to this controversy, here we refer to phenotypic variation, a more general and less-controversial term.

In our lab, we investigate different aspects of the Gram-negative bacterium *Acidovorax citrulli*, the causal agent of bacterial fruit blotch (BFB) disease of cucurbit plants [20–22]. BFB started to gain importance during the late 1980s’ after devastating outbreaks in watermelon fields in the USA. Since then the disease spread to many parts of the world and to other cucurbit crops, including melon, pumpkin, squash and cucumber [23,24]. All aerial parts of the host plants are infected by the bacterium; however, young seedlings and fruits are extremely susceptible. Due to the lack of efficient methods to control BFB, this disease represents a serious threat to the cucurbit industry, and primarily of melons and watermelon [23–25].

*A. citrulli* strains can be divided into at least two well-differentiated groups: group I includes strains that were mainly isolated from melon and other non-watermelon cucurbit plants, while group II includes strains that cause typical BFB in watermelon. These groups can be distinguished by carbon substrate utilization, fatty acid methyl ester analysis, DNA-fingerprinting and virulence on different hosts. In addition, group I strains are moderately to highly aggressive on a wide range of cucurbits, while group II strains are highly aggressive in watermelon but only mildly so on other cucurbits [26–28]. One group II strain, AAC00-1, has been sequenced by the Joint Genome Institute and its sequence is available to the scientific community (GenBank accession NC_008752). Nevertheless, and despite the economic importance of BFB, still little is known about virulence and adaptation mechanisms of *A. citrulli* as well as basic aspects of *A. citrulli*-host interactions. In this regard, we have shown that *A. citrulli* relies on a functional type III secretion system (T3SS) to promote disease [23], and that type IV pili (T4P) and polar flagella are important virulence determinants of this pathogen [29–31].

We have recently detected the phenomenon of phenotypic variation in *A. citrulli* strains belonging to both groups I and II. Here we report this phenomenon and describe the characterization of phenotypic variants (PVs) in terms of morphological and metabolic traits, motility, biofilm formation and virulence.

## Materials and Methods

### Bacterial strains and growth conditions

*Acidovorax citrulli* strains used in this study are listed in Table 1. The strains were grown in NB (Difco Laboratories, Detroit, MI, USA) or NA (NB containing 15 g/l agar) at 28°C, unless stated otherwise. For plant inoculation, strains were grown on NA for 48 h, resuspended from plates in sterile distilled water (SDW), adjusted to an OD_600_ of 0.6 [about 10^8^ colony forming units (CFU)/ml] using a Helios Gamma spectrophotometer (Thermo Electron Corp., Rochester, NY, USA), and then diluted to the desired concentration.

**Table 1:** *Acidovorax citrulli* strains used in this study.

**Strain**	**Characteristics**	**Reference / source**
M6	Group I wild-type strain isolated in Israel from a melon fruit in 2002, Ap^r^	[25]
7a1	Group II wild-type strain isolated in Israel from a watermelon plant in 1996, Ap^r^	Kindly provided by I. Assouline
M6V1	Phenotypic variant of M6 isolated after melon cotyledon inoculation, Ap^r^	This study
M6V2	Phenotypic variant of M6 isolated after exposure to pH stress (pH 5.8), Ap^r^	This study
7a1V1	Phenotypic variant of 7a1 isolated after exposure to pH stress (pH 5.8), Ap^r^	This study
7a1V2	Phenotypic variant of 7a1 isolated after exposure to salt stress (100 mM NaCl), Ap^r^	This study
M6-2-23	Transposon mutant of strain M6 impaired in gene *pilM*	[28]
M6-2g-84	Transposon mutant of strain M6 impaired in gene *pilM*	This study

Ap^R^, resistance to ampicillin

### Isolation of phenotypic variants

*A. citrulli* M6 and 7a1 were grown in NB at 28°C with shaking (150 rpm) for 24 hours. Then cells were collected and washed twice with 100 mM phosphate buffer (pH 7.0). Collected bacteria were exposed to three different stresses: salt, acidic pH and high temperature. Salt stress consisted of exposure to 100 mM NaCl for 24 h. Acidic pH stress consisted of exposure to pH 5.8 in 100 mM phosphate buffer for 4 h. High temperature was done by exposure of cells to 45°C for 1 h. In addition, phenotypic variants (PVs) were isolated from artificially inoculated plants. For these experiments, one-week-old melon (cv. Ofir, Zeraim Gedera, Israel) and watermelon (cv. Malali, Hazera Genetics, Israel) seedlings were inoculated with strains M6 and 7a1, respectively. Seedling cotyledons were scratched and bacterial suspensions with 10^8^ CFU/ml were swabbed on the ruptured tissue. The seedlings were kept in a greenhouse at 27-28°C and bacteria were isolated from the inoculated tissue after 7 days. After these treatments, bacteria were collected and serially diluted in SDW. Dilutions were plated onto NA plates (in the case of isolations from plant tissues, the media also contained 100 µg/ml ampicillin). Colony morphology was assessed after 3 days of incubation at 28°C and the percentage of appearance of altered (variant) colonies was determined.

### Microscopy

Morphological observations of bacterial colonies were done using a SMZ1500 stereomicroscope (binocular) equipped with a DS-Ri1 color digital camera (both from Nikon, Tokyo, Japan). For scanning electron microscopy (SEM), cells were grown on NA plates. Then colonies were excised with a thin slice of agar block. For fixation, these pieces were dipped into 4% glutaraldehyde, prepared in 100 mM phosphate buffer (pH 7.0) for 1 h. They were then washed three times with the same buffer and dipped into 1% osmium tetraoxide prepared in 100 mM phosphate buffer (pH 7.0). The blocks were then rinsed and dehydrated using increasing ethanol concentrations of 25%, 50%, 75%, 95% and 100%. The blocks were then dried in a Critical Point Dryer (CPD 030; Bal-Tec, Schalksmühle, Germany) displacing the alcohol with liquid CO_2_ and finally dried by releasing CO_2_. The dried blocks were mounted on the brass blocks, coated with gold (Polaron Spatter Coater) and visualized with a JSM-5410LV scanning electron microscope (Jeol Ltd., Tokyo, Japan) under vacuum. Cells were grown similarly for transmission electron microscopy (TEM). Following growth, a 5-µl drop of SDW was loaded onto each selected colony for 2 min. Then a 300-mesh formvar-coated carbon grid was placed on the top of each colony for 1 min to adsorb the colony into the grid. For negative staining, the grids were placed into drops of 1% uranyl acetate for 10 s. Liquid excess was removed by soaking the drop from the side with a Whatman paper. The grids were then dried and observed using a Tecnai-12 electron microscope (Philips Electron Optics, Eindhoven, Netherlands) equipped with a Megaview II CCD camera and AnalySIS version 3,0 software (Soft Imaging System GmbH, Munster, Germany). For assessment of twitching motility, bacteria were grown on NA plates for 72 h at 28°C. Then, colony edges were observed under a Nikon Eclipse 80i microscopy (X10 magnification) equipped with a DS-Qi1Mc digital camera and the NIS elements software (Nikon). Images were captured every 15 min for a period of 6 h. Time lapse movies were created using the ImageJ software (NIMH, Bethesda, MD, USA).

### Swimming motility assays

These assays were performed as described [29] with few modifications. Briefly, PVs and parental strains were grown on NA plates for 48 h. Then, cells were resuspended in SDW and adjusted to OD_600_ ~0.5. Two microliters of bacterial suspensions were transferred to the center of soft (0.3% agar) NA plates. The plates were incubated at 28°C and digital pictures of the haloes formed by migrating bacteria were taken after 24 h with a SP-590 UZ Olympus digital camera (Olympus, Center Valley, PA, USA). Halo areas were measured using APS Assess software (American Phytopathological Society, St. Paul, MN, USA).

### Biofilm formation assays

Biofilm assays were performed as described [29] with few modifications. Overnight cultures of bacterial strains were prepared in NB. The cultures were diluted in a 1:100 ratio with fresh XVM2 medium [32]. Two hundred microliters of the diluted suspensions were poured into wells of 96-well polystyrene microplates (Nunc, Roskilde, Denmark) and incubated at 28°C for 48 h. Then media were discarded and the wells were rinsed twice with SDW. Wells were then refilled with 250 µl of 0.1% methyl violet-B and incubated at room temperature for 30 min. The solution was discarded, the wells were washed twice with SDW and refilled with 300 µl of 95% ethanol. After 2 hours, 250 µl of the suspensions of each well were collected, transferred to new 96-well plates, and the optical densities of the suspensions were measured at 595 nm using an Infinite F200 ELISA plate reader (Tecan Group Ltd., Männedorf, Switzerland).

### Virulence assays

PVs were compared with parental strains for their virulence by seed transmission assays, which were carried out as described [29] with few modifications. Briefly, 24 h-old cultures of bacterial strains, grown on NA, were resuspended in 10 mM MgCl_2_, with suspensions being adjusted to bacterial concentrations of 10^6^ CFU/ml. Then, groups of 16 seeds of melon (*Cucumis melo* cv. Ophir; Zeraim Gedera, Gedera, Israel) and watermelon (*Citrullus lanatus* cv. Malali; Hazera Genetics, Gedera, Israel) were incubated with 15 ml of each bacterial suspension for 2 h, at room temperature with gentle agitation. Melon seeds were inoculated with M6 parental strain or variants, while watermelon seeds were inoculated with 7a1 parental strain or variants. Seeds were collected, briefly air-dried under a laminar flow, and sown individually in plastic poly-cups filled with a peat-based commercial soil mixture (Shacham Gival Ada, Givat Ada, Israel). Seedlings were grown for 14 days in a greenhouse at 27-28°C, and during this period, we followed daily after symptom development and seedling death.

### Omnilog GEN III MicroPlate assays

The metabolic characteristics of parental strains and PVs were analysed using GEN III Microplates (Biolog Inc., Hayward, CA, USA). This method analyzes the respiration kinetics of a microorganism based on 94 phenotypic tests including 71 sole carbon sources and 23 chemical sensitivity assays, thus providing a phenotype fingerprint of the tested microorganism. Increased respiration causes formation of a purple color through reduction of the tetrazolium redox dye, indicating metabolic activity. Cells grown on LB agar plates (10 g/l peptone, 5 g/l yeast extract, 10 g/l sodium chloride, 15 g/l agar; pH 7.5) for approximately 20 hours were used to inoculate the microplates with a cell suspension in IF-A inoculation fluid at a cell density of 95% transmission. Each strain was studied in three independent repetitions. Briefly, the microtiter plates with substrates, dye, and bacterial cells are loaded into the OmniLog® reader, a hardware device which provides the appropriate incubation conditions (at 28°C for 96 hours) and also automatically reads the intensity of color formation during tetrazolium reduction every 15 min. The exported measurement data were further analysed with the opm package for R [33,34], using its functionality for displaying the curve kinetics and aggregating curve parameters such as the maximum value (A) and the area under the curve (AUC) by spline fitting algorithms. The functionalities of the opm package are described in detail at http://cran.r-project.org/web/packages/opm/vignettes/opm.pdf.

### Pulsed-field gel electrophoresis (PFGE)

Bacteria were grown on NA at 28^°^C for 48 h. Cells were suspended in a buffer containing 100 mM EDTA and 100 mM NaCl (pH 8.0), and adjusted to an OD_600_ of 0.3. Bacterial suspensions (1.5 ml) were pelleted at 10,780g for 2 min and resuspended in 300 µl of the same buffer, followed by the addition of 60 µl of a 10 mg/ml lysozyme solution. Preparation of agarose plugs was as described [27]. Plugs were transferred to 1.5 ml Eppendorf tubes and 140 µl restriction mixtures were added to each tube. Four enzymes were used individually for digestion of the DNA: SpeI, NotI, XbaI and HindIII. The restriction mixture of SpeI was prepared with 15 µl of SpeI 10Xbuffer (NEB, Ipswich, MA, USA) 1.5 µl of 0.01 mg/ml BSA (NEB), 2 µl of SpeI (10U/µl; NEB) and 121.5 µl of sterile double distilled water (sDDW). The restriction mixture of NotI was prepared with 15 µl of 10Xbuffer 4 (NEB), 1.5 µl of 0.01 mg/ml BSA, 2 µl of NotI (20 U/µl; NEB) and 121.5 µl of sDDW. The restriction mixture of XbaI was prepared with 15 µl of 10Xbuffer Tango (Fermentas, Ontario, Canada), 2 µl XbaI (10U/µl; Fermentas) and 121.5 µl of sDDW. The restriction mixture of HindIII was prepared with 15 µl of 10Xbuffer Tango, 1.5 µl of buffer R (Fermentas), 2 µl of HindIII (10 U/µl; Fermentas) and 121.5 µl of sDDW. Digestions were performed at 37°C for 24 hours. PFGE was carried out in 15x15 cm 1% D5 agarose (Hispanagar, Burgos, Spain) gels using a CHEF DR–II apparatus (Bio-Rad, Hercules, CA, USA). The gels were run in 0.5X TBE buffer at a temperature of 14°C with initial switch time of 5 s, final switch time of 45 s, 4.5 V/cm and 22 hour running time, as described [27]. Concatemeric lambda DNA (NEB) was used as a marker. Following electrophoresis, gels were stained in an ethidium-bromide solution (0.5 µg/ml) and photographed with transmitted UV light at 295 nm.

### Repetitive-PCR (rep-PCR)

For DNA extraction, bacteria were grown in NB at 28^°^C for 24 h. DNA was extracted using the GeneElute^TM^ Bacterial Genomic DNA Kit (Sigma-Aldrich, St. Louis, MO, USA). Primers REP1R-I and REP2-I (for repetitive extragenic palindrome-PCR; REP-PCR), ERIC1R and ERIC2 (for enterobacterial repetitive intergenic consensus-PCR; ERIC-PCR) and BOXA1R (for BOX element-PCR; BOX-PCR) were as described by Louws et al. [35], and were purchased from Hy Labs (Rehovot, Israel). The PCR mixtures (25 µl) contained: 1 U of Red Taq Polymerase (Sigma), 2.5 µl 10X buffer (Sigma), 2.5 µl of 25 mM MgCl_2_, 0.2 µM of each primer (0.4 µM of BOXA1R in BOX-PCR), 0.4 mM of each dNTP (Promega, Madison, WI, USA), 2.5 µl of 20 mg/ml bovine serum albumin (Roche, Basel, Switzerland) and 1 µl (20-30 ng) of template DNA. Amplifications were performed in an Eppendorf Thermal Cycler (Eppendorf, Hamburg, Germany) with an initial denaturation cycle of 7 min at 95°C, followed by 30 cycles of denaturation for 1 min at 94°C, annealing for 1 min at 40, 42 or 53°C (for REP-, ERIC- and BOX-PCR, respectively), and elongation at 65°C for 8 min. A final extension step was performed at 65°C for 16 min. Samples of 15 µl from each reaction were run in 1.5% agarose gels for 2 h at 6 V/cm. The gels were stained with an ethidium-bromide solution (0.5 µg/ml) and photographed with transmitted UV light at 295 nm.

### Statistical analyses

The R package multcomp [36] was used in order to infer significant differences and their 95% confidence intervals on pairwise comparisons of the parental strains and their PVs for the following datasets: CFU/colony, swimming motility, biofilm formation and metabolic traits using respiration kinetics from Omnilog GEN III microplates. Multcomp allows for simultaneous pairwise comparisons of the means of multiple groups under control of the familywise error rate. The familywise error rate is the probability of falsely rejecting one or more hypothesis (i.e. finding a significant difference among the means of any two groups in the dataset even though there is actually no difference present). The familywise error rate increases with the number of groups that are compared pairwise to each other. Multcomp is especially suited for data with unequal variances and a non-normal distribution [37]. All details on the statistical and graphical analyses of the above addressed datasets are documented in Appendix S1.

## Results

### *Acidovorax citrulli* exhibits the phenomenon of phenotypic variation

During routine investigation in our lab, we have observed phenotypic variation in *A. citrulli* strains M6 and 7a1, belonging to group I and II, respectively. Bacterial colonies of these strains with altered morphology relative to their corresponding parental strains were observed at variable frequencies following standard growth in rich medium (nutrient agar, NA), as well as upon isolation of bacteria from inoculated plants and exposure to several stresses, including heat (exposure to 45 ^°^C for 1h), salt (100 mM NaCl for 24 h) and acidic conditions (pH 5.8 for 4 h). The percentage of appearance of phenotypic variant (PV)-type colonies relative to the total number of colonies varied for the same treatment among multiple experiments, ranging from non-detected (the minimum level of detection in our experimental set-up was of about 0.01%) to 1.5%. It also appeared that among the aforementioned treatments, exposure to acidic conditions promoted highest frequencies of phenotypic variation, since PVs of both strains were always detected after exposure to this treatment, and the frequencies of appearance of PV colonies were the highest, ranging from 1.0 to 1.5% in strain M6, and 0.3 to 0.8% in strain 7a1. To confirm that the morphologically altered colonies are PVs of *A. citrulli* and not contaminations, many PV suspected colonies were tested with *A. citrulli* specific PCR primers BX-S [38]. The results were always positive (not shown), thus confirming that these indeed are *A. citrulli* PVs.

**Figure 1:**
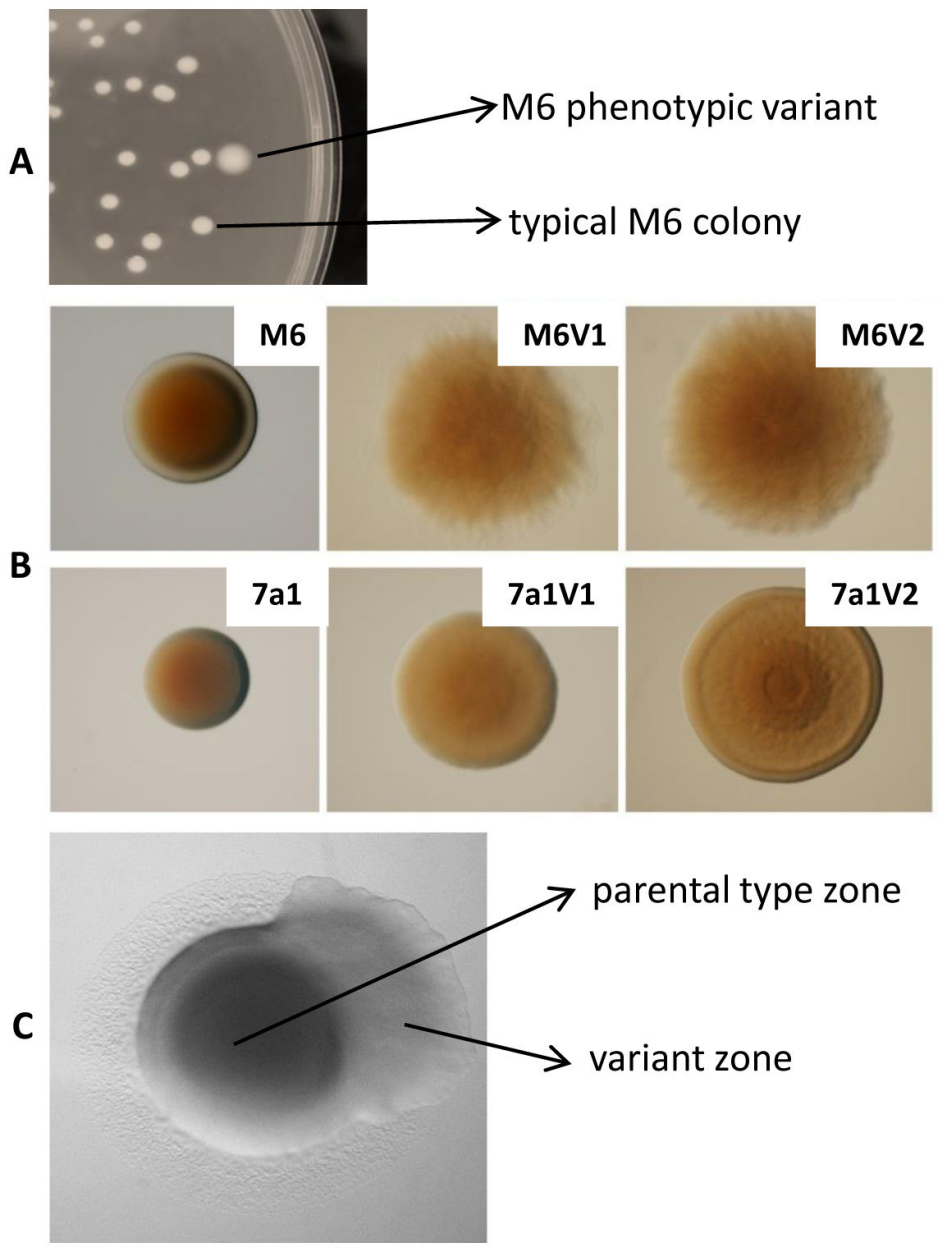
Phenotypic variation in *Acidovorax citrulli*. **A**. A phenotypic variant (PV) colony of strain M6 after 72 h of growth in NA. PV colonies of strain 7a1 have a similar appearance when observed at the naked eye. **B**. Binocular observation of 72-h colonies (grown on NA) of parental strains M6 and 7a1 and two variants of each strain, M6V1 and M6V2, and 7a1V1 and 7a1V2 (the same magnification was used for all colonies). **C**. Phenotypic variation during growth of an *A. citrulli* M6 colony.

When grown on NA plates, the colonies of all PVs of strains M6 and 7a1 were shown to possess a translucent appearance, in contrast to the parental strain colonies that are opaque (Figure 1A). After 72 h of growth, PV colonies were also shown to be bigger than the parental colonies (Figure 1A). Binocular observations revealed that PV colonies have a fuzzy appearance relative to those of the parental strains that are relatively smooth (Figure 1B). This phenotype was more pronounced in M6 than in 7a1 variants. Some morphological differences could also be observed between 7a1 variants; for instance variant 7a1V2 formed concentering rings that were absent in the parental strain and in variant 7a1V2 (Figure 1B).

**Figure 2:**
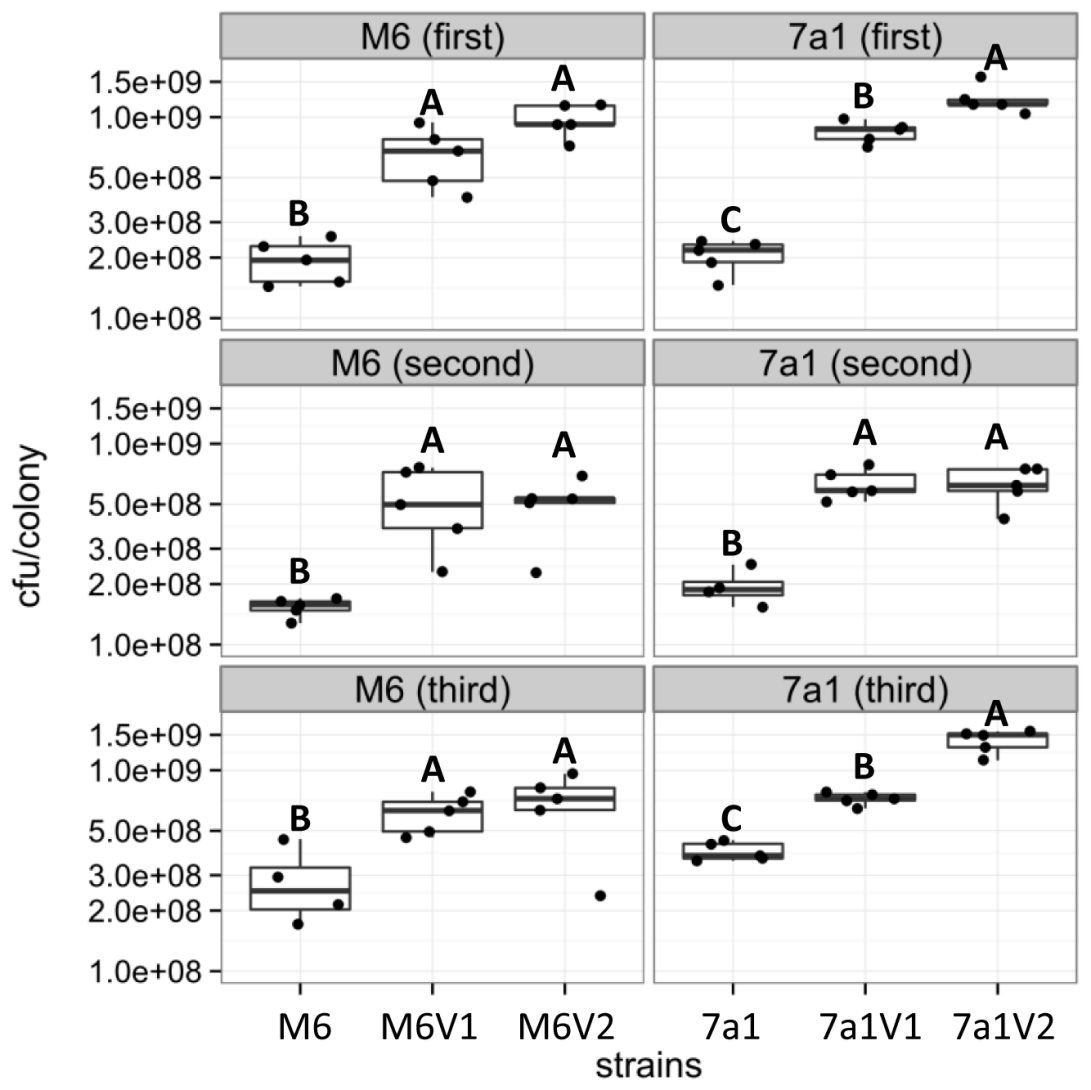
Estimation of colony forming units (CFU) per colonies of strains M6 and 7a1 and their corresponding variants, after 72 h of growth on NA. CFU/colony was determined following serial dilution plating of five colonies per strain. Top, middle, and bottom panels show the results of three independent experiments (first, second, third) for each strain and corresponding variants (5 replicates per strain per experiment). Each dot indicates the CFU of a single colony. The box-and-whisker plots indicate minimum, first quartile, median, third quartile, and maximum values. Different letters indicate statistically significant differences (p < 0.05; for details see Appendix S1) among strains in each experiment.

Phenotypic variation may also occur during growth of a single colony on NA plates under standard conditions. In this case, the colony has a mixed appearance (shown for strain M6 in Figure 1C). Note that the parental type zone is surrounded by a clear halo, while the variant zone is not. Haloes around typical *A. citrulli* colonies are formed as result of type IV pilus (T4P)-mediated twitching motility, and they are clearly observed by the naked eye after 72 h of growth on NA [29]. The lack of halo formation around PV colonies, or PV zones in mixed colonies, could be explained by enhanced growth of the PVs relative to the parental strains on NA plates, and/or lack of T4P activity in the variants. To the second possibility we will refer later on in the results’ description. In terms of growth ability, indeed quantification of CFU per colony in two variants of each strain after 72 h of growth on NA revealed that PV colonies possess significantly higher (p < 0.05) number of cells than colonies of the parental strains (Figure 2, Appendix S1). This finding may also explain, at least partially, the increased size of the PV colonies relative to parental strain colonies. Scanning electron microscopy (SEM) observations did not reveal substantial differences in cell size and morphology between parental and variant cells, although PV cells possessed slightly increased roughness than parental strains (shown in Figure S1 for strain M6 and two of its variants).

### PVs differ from their parental strains in swimming motility and biofilm formation ability

**Figure 3:**
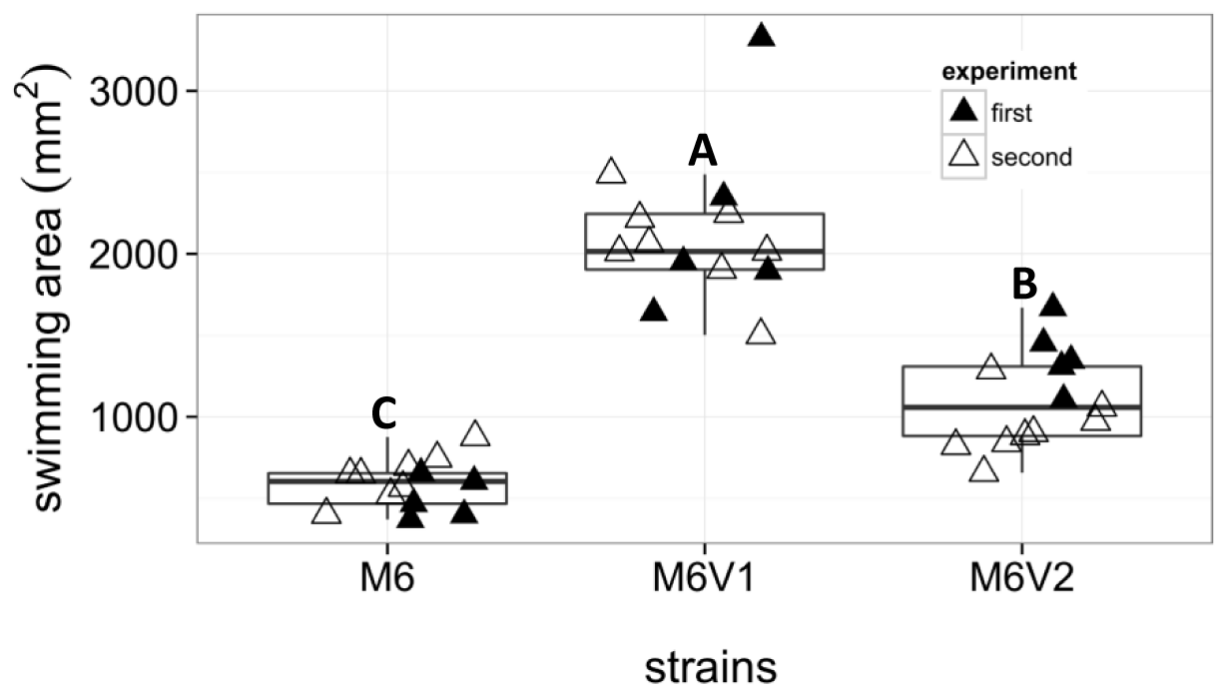
Phenotypic variants M6V1 and M6V2 possess increased swimming motility than the parental strain. Swimming assays were performed on soft NA plates (0.3% agar). Swimming areas were measured after 24 hours of incubation at 28°C. Each triangle represents a replicate from two independent experiments (first and second experiment replicates, represented by black and white triangles, respectively). The box-and-whisker plots indicate minimum, first quartile, median, third quartile, and maximum values, summarized from the two experiments. Different letters indicate statistically significant differences (p < 0.001; for details see Appendix S1) among strains.

Light microscopy observations of cells from liquid cultures revealed that variants of strain M6 appear to possess higher swimming motility than the parental strain. Variants M6V1 and M6V2 were selected for swimming motility assays on semi-solid NA plates containing 0.3% agar. Swimming assays confirmed that these PVs possess significantly (p < 0.001) higher swimming motility than parental strain M6, while variant M6V1 had significantly increased (p < 0.001) swimming motility than variant M6V2 (Figure 3, Appendix S1). As opposite as to the picture observed for M6 variants, PVs of strain 7a1 were characterized by the lack of swimming motility as observed in light microscopy observations. Swimming motility assays of two variants- 7a1V1 and 7a1V2- on soft agar NA plates confirmed these results (Figure S2). In agreement with these results, we were not able to detect polar flagella in SEM and TEM observations of 7a1 PVs (not shown).

**Figure 4:**
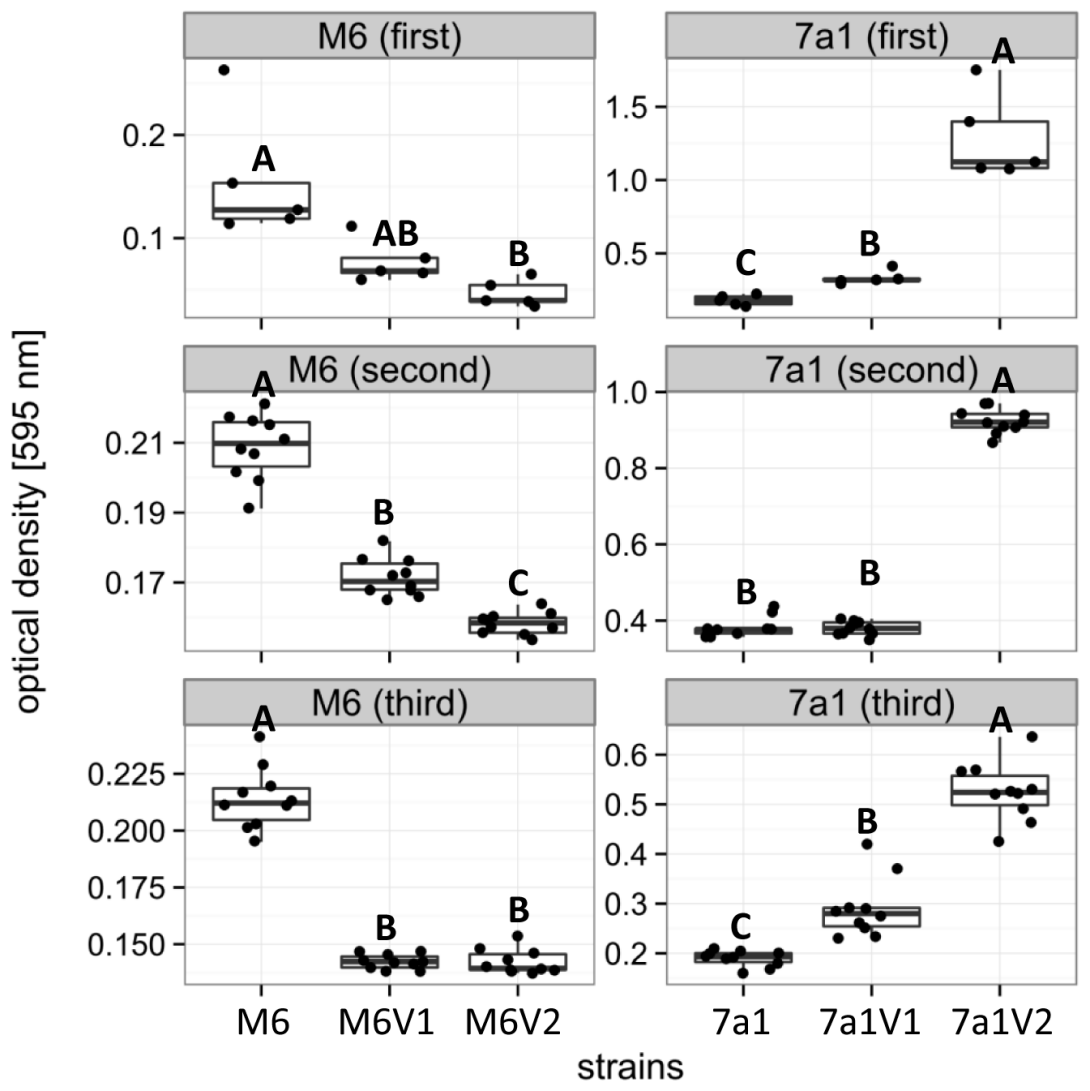
Biofilm formation ability of strains M6 and 7a1 and their corresponding variants. Cultures were grown in XVM2 medium for 48 hours in polystyrene ELISA plates at 28 ^°^C. Formed biofilms at the medium/air interface were stained with 0.01% crystal violet stain. Stained biofilms were resuspended with ethanol and quantitatively estimated by optical density measurements at 595 nm. Top, middle and bottom panels show the results of three independent experiments (first, second, third; with five replicates per strain in the first experiment, and ten replicates per strain in second and third experiments). The box-and-whisker plots indicate minimum, first quartile, median, third quartile, and maximum values. Different letters indicate statistically significant differences (p < 0.05; for details see Appendix S1) among strains in each experiment.

We also compared between PVs and parental strains for their biofilm formation ability by standard biofilm assays in polystyrene culture plates. Figure 4 shows that variants M6V1 and M6V2 possess significantly (p < 0.05) reduced biofilm formation ability than their parental strain M6. In contrast, variants 7a1V1 and 7a1V2 were shown to have significantly (p < 0.05) enhanced biofilm formation ability than their parental strain (Figure 4, Appendix S1).

### PVs of both strains are reduced in virulence relative to the parental strain

**Figure 5:**
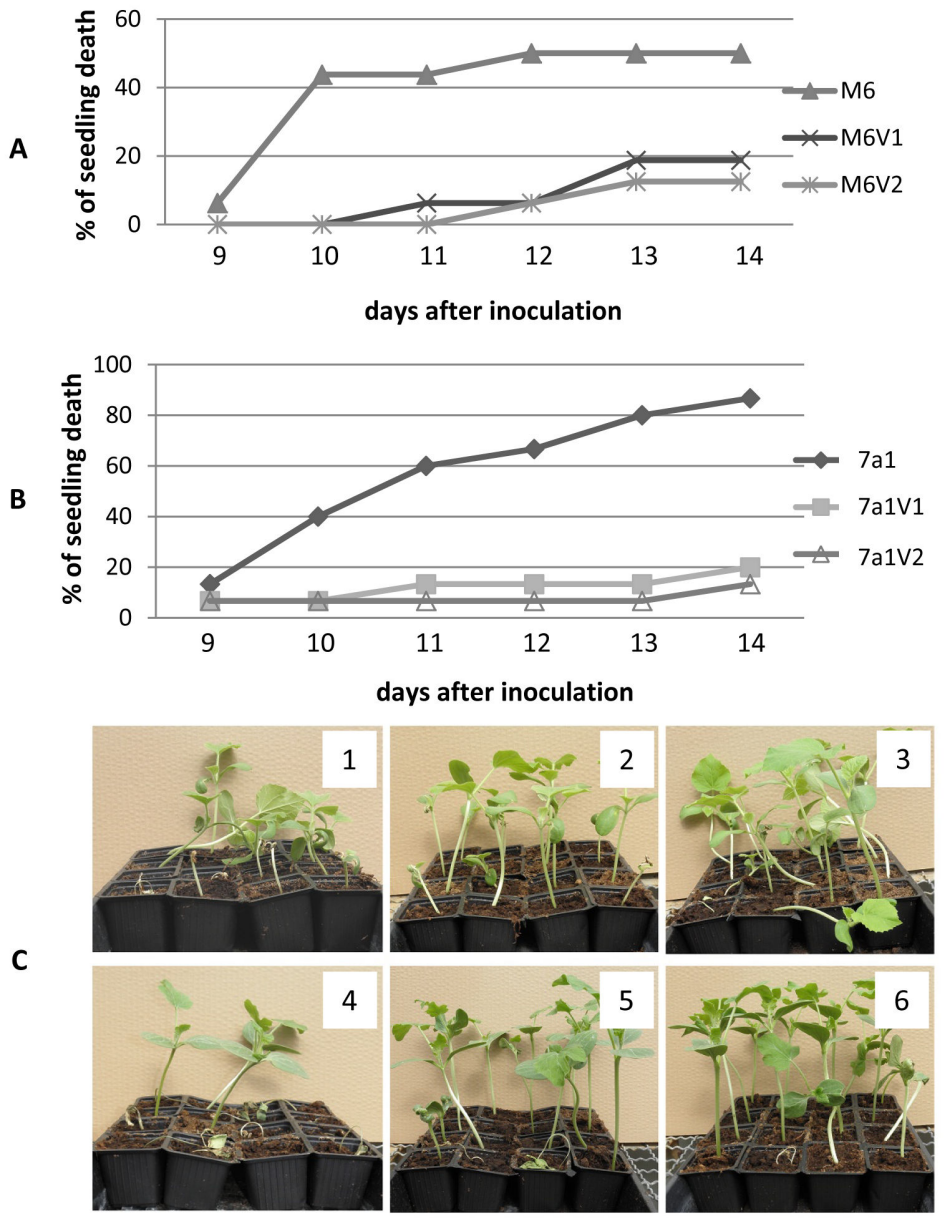
PVs of strain M6 and 7a1 are compromised in their virulence in seed transmission assays. Melon or watermelon seeds were inoculated with bacterial strains at 10^6^ CFU/ml, sown and maintained in the greenhouse at 28°C for 14 days. The percentage of seedling death was determined thorough the experiment. **A**. Melon seedlings following inoculation with parental strain M6 and variants M6V1 and M6V2. **B**. Watermelon seedlings following inoculation with parental strain 7a1 and variants 7a1V1 and 7a1V2. For each combination of plant/strains, data from one experiment (with 16 plants per treatment), out of three with similar results are shown. **C**. Symptom severity of melon (1 to 3) and watermelon (4 to 6) plants from seed transmission assays with parental strains and PVs. 1, M6; 2, M6V1; 3, M6V2; 4, 7a1; 5, 7a1V1; 6, 7a1V2. Pictures were taken 12 days after inoculation.

To assess whether PVs of M6 and 7a1 are altered in their virulence relative to the parental strains, we performed seed transmission assays on melon and watermelon, respectively. These experiments revealed that all tested variants were compromised in their ability to induce seedling blight and wilt symptoms in comparison with their parental strains. In the case of strain M6, while 50% of seedlings inoculated with the parental strain died 12 days after inoculation (d.a.i.), the percentage of seedling death induced by variants M6V1 and M6V2 at the same time was below 10%, and it did not increase over 20% after 15 days (Figure 5A). In the case of 7a1, while inoculation with the parental strain led to death of over 80% of watermelon seedlings 14 d.a.i., the percentage of seedling death following inoculation with the variants was of 15-20% by the same time (Figure 5B). Pictures of symptomatic plants for all treatments from one of the experiments are shown in Figure 5C.

### M6 and 7a1 variants do not produce T4P during growth on NA

**Figure 6:**
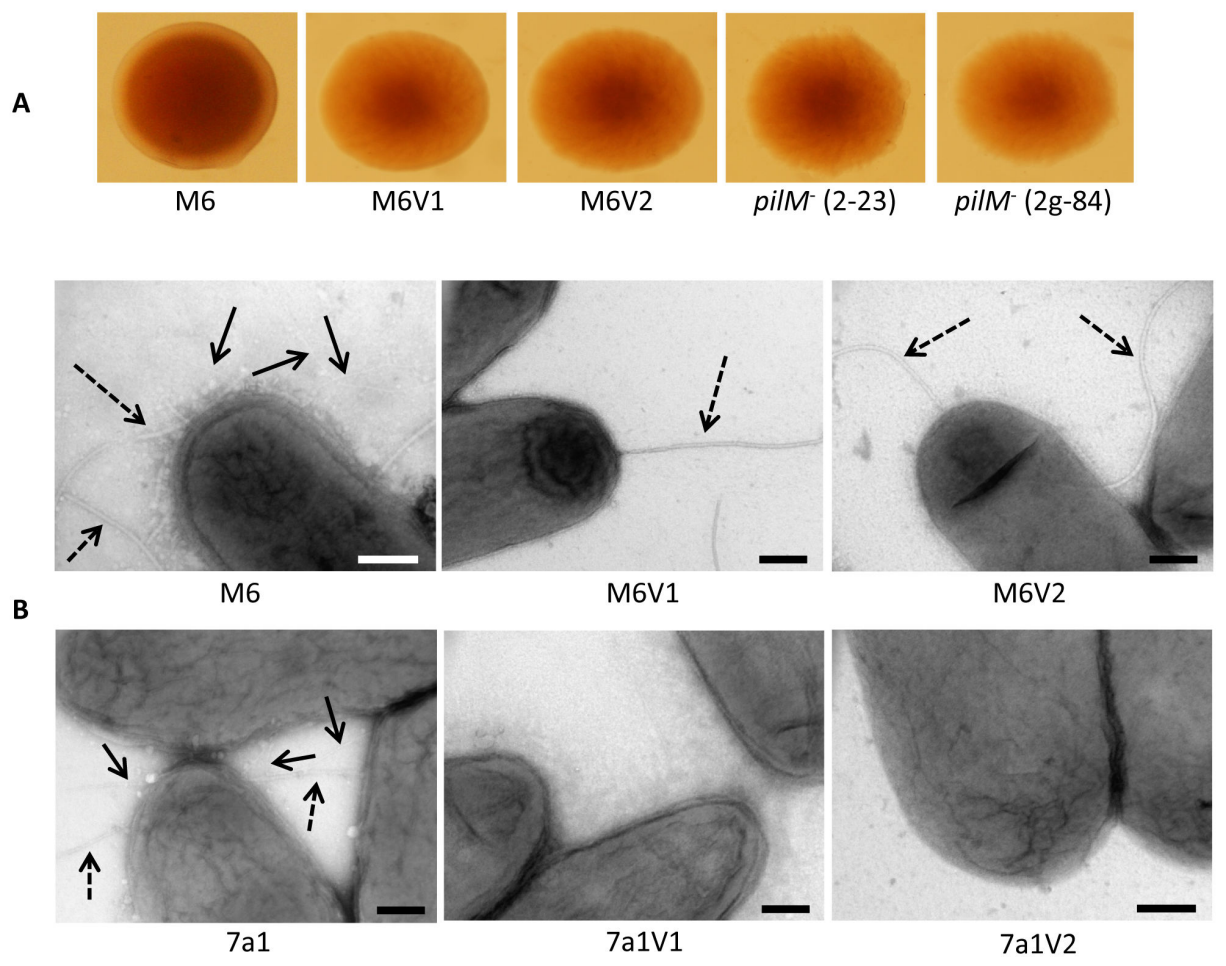
PVs of *A. citrulli* lack the ability to produce type IV pili (T4P) in NA medium. **A**. Binocular observation of 72-h colonies of parental strains M6, variants M6V1 and M6V2 and two T4P (*pilM* ^-^) mutants of strain M6, M6-2-23 and M6-2g-84. **B**. Transmission electron microscopy of parental strains M6 and 7a1, and PVs of both strains, following growth for 72 h on NA plates. Solid and dashed arrows indicate T4P and polar flagella, respectively. No T4P could be detected in PVs of all variants and no polar flagella could be detected in PVs of strain 7a1. Bars, 200 ηm.

We have recently characterized mutants of *A. citrulli* M6 lacking the ability to produce T4P [29,30]. Interestingly, the *A. citrulli* PVs characterized here showed some phenotypic similarities with T4P mutants of this bacterium. These include colony morphological similarities in terms of colony appearance and increased colony size relative to wild-type strain, reduced virulence, increased swimming motility and reduced biofilm formation ability (the last two features, in the case of T4P mutants and PVs of strain M6). Binocular observations confirmed that colonies of two M6 *pilM* ^-^ mutants available in our lab collection (Table 1) have a similar appearance than those of M6 PVs (Figure 6A). Moreover, as similar as previously shown for M6 *pilM* ^-^ mutants, transmission electron microscopy (TEM) observations revealed that both M6 and 7a1 variants analyzed in this study do not have the ability to form T4P under tested conditions (Figure 6B), thus explaining the absence of twitching haloes around colonies of the variants. In addition, in agreement with the findings showing that the characterized 7a1 PVs lack swimming ability, no polar flagella could be detected in TEM observations of these PVs (Figure 6B). In an additional approach to verify the lack of twitching motility in the PVs, we generated time-lapse movies focusing on the edges of parental strain and PV colonies grown on NA plates for a period of 6 h. As expected, twitching zones and twitching motility could be detected at the colony edges of parental strains M6 and 7a1 (movies S1 and S2, respectively). In contrast, these could not be detected in variants M6V1, M6V2, 7a1V1 and 7a1V2, with the expansion of colony edges of the variants being due to colony growth (shown for variants M6V1 and 7a1V1 in movies S3 and S4, respectively), as similar as observed for the M6 *pilM* ^*-*^ mutant M6-2-23 (movie S5), which is known to lack twitching motility [29].

### Variants differ from parental strains in the dynamics of utilization of few carbon sources

PVs were compared with parental strains M6 and 7a1 by OmniLog (Biolog) GEN III MicroPlate analysis. This method is commonly used for bacterial species/subspecies identification, thus as expected, PVs and parental strains did not differ in tetrazolium reduction patterns in most plate wells. However, few differences could be observed. For each of the below addressed carbon sources (see also Figure S3), at least one of the pairwise comparisons among parental strain and variants was statistically significant (see also the statistical details in Appendix S1). For instance, M6V1 and M6V2 showed slightly enhanced utilization of D-fructose, D-saccharic acid and α-hydroxyl-butyric acid relative to parental strain M6 (Figure S3A, Appendix S1). On the other hand, variants 7a1V1 and 7a1V2 showed slightly enhanced utilization of mucic acid and D-lactic acid methyl ester than parental strain, and slightly reduced utilization of D-threalose. Variant 7a1V2 showed reduced utilization of D-glucuronic acid, γ-amino-n-butyric acid and increased sensitivity to sodium formate than the parental strain and variant 7a1V1 (Figure S3B, Appendix S1).

### Comparison among PVs and parental strains by DNA fingerprinting techniques

Phenotypic variation can be associated with large DNA rearrangements. In such cases, PVs can be distinguished from their parental strains by DNA fingerprinting methods [13,39–41]. We assessed whether variants M6V1 and M6V2 as well as 7a1V1 and 7a1V2 differ from their corresponding parental strains, in pulse field gel electrophoresis (PFGE) and repetitive-PCR (rep-PCR) profiles.

**Figure 7:**
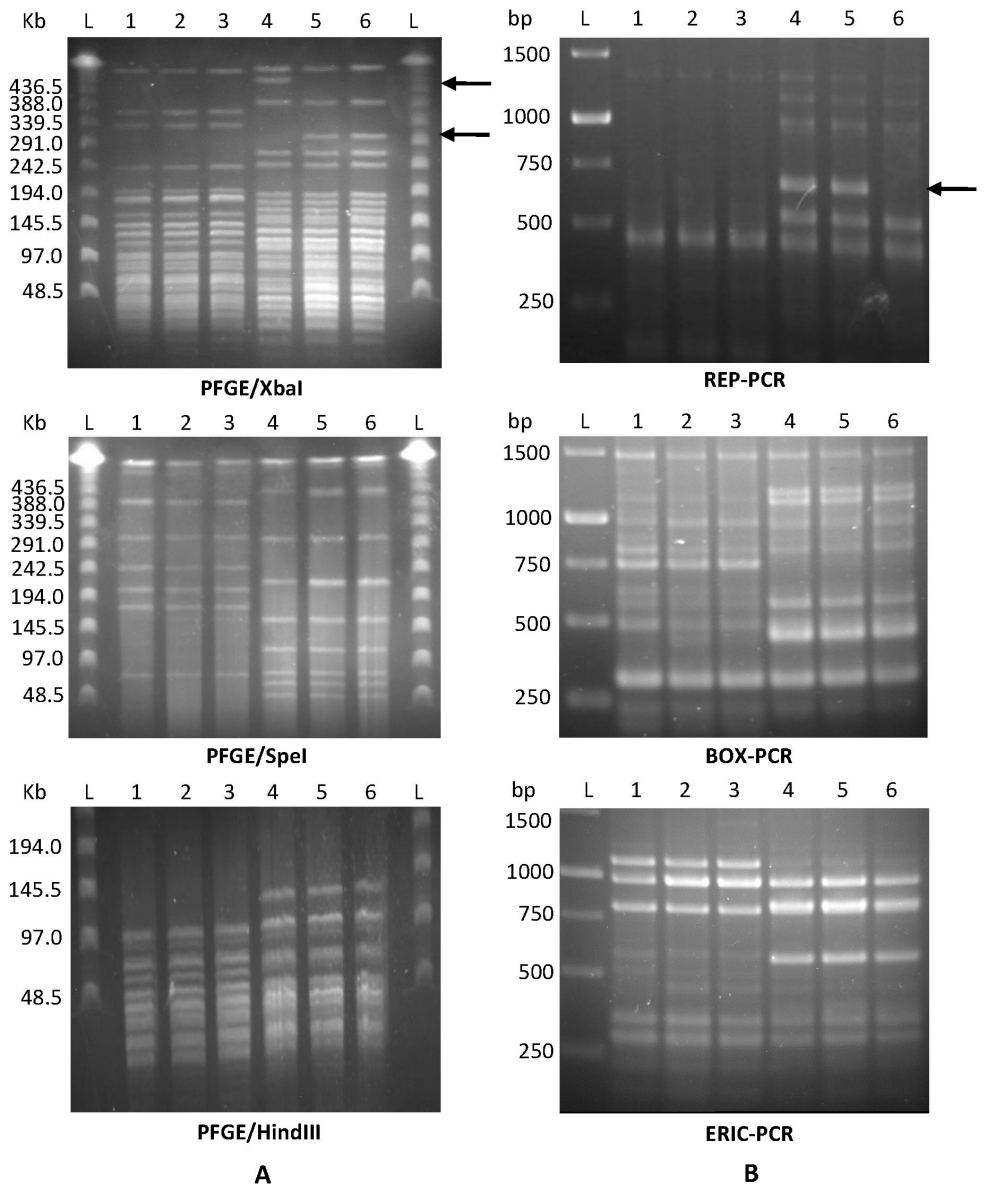
DNA fingerprint profiles of strains M6 and 7a1, and their variants. **A**. Pulse field gel electrophoresis (PFGE) following digestion with XbaI, SpeI and HindIII. **B**. repetitive-PCR profiling by REP-, BOX- and ERIC-PCR. Lines: **L**, ladder; **1**, M6; **2**, M6V1; **3**, M6V2; **4**, 7a1; **5**, 7a1V1; **6**, 7a1V2. Arrows indicate differences between some of the variants and their parental strain. Results are representative from at least two independent runs with similar results for each method.

No differences were observed between PVs of strain M6 and the parental strain in PFGE following digestion with restriction enzymes XbaI, SpeI, HindIII and NotI (shown for the first three in Figure 7A), as well as in three rep-PCR tests (BOX-, ERIC- and REP-PCR (Figure 7B). These results suggest that in *A. citrulli* M6, phenotypic variation is associated with slight genetic modifications or epigenetic changes, which are difficult to detect using these fingerprinting approaches.

In contrast to strain M6, some differences could be distinguished between parental strain 7a1 and its PVs in some of these comparisons. While PVs of strain 7a1 did not differ from the parental strain in PFGE following restriction with SpeI, HindIII or NotI, they did slightly differ from the parental strain when XbaI was used (Figure 7A). No differences were found between strain 7a1 and its PVs in BOX- and ERIC-PCR. However, one of the variants, 7a1V2, differed in its REP-PCR profile from the parental strain and from variant 7a1V1: a ~700-bp band present in the parental strain and in variant 7a1V1 was absent in the REP-PCR profile of variant 7a1V2 (Figure 7B).

## Discussion

Phenotypic variation is a well-recognized phenomenon in plant-associated Gram-negative bacterium [13,41–45]. Essentially, it is an adaptive mechanism that may lead to increased fitness and survival of an organism, depending upon its ability to colonize, persist and spread around the environment. Phenotypic variation results in intra-population diversity, in which a minority of the population has an altered phenotype. It is assumed that this monitory may not possess a selective advantage under the current conditions; however, under altered conditions, it can become dominant if they have a selective advantage over the parental type [46].

Despite accumulated knowledge about phenotypic variation in environmental and animal pathogenic bacteria, still there is a major gap in our understanding whether this phenomenon has a major impact on bacterial fitness [10]. This is particularly true in the case of plant pathogenic bacteria for which phenotypic variation has not been deeply investigated. Here we reported the occurrence of phenotypic variation in the plant pathogenic bacterium *Acidovorax citrulli*. To the best of our knowledge, this is the first report of phenotypic variation of a plant pathogenic *Acidovorax* species.

In this study we were able to detect many phenotypic variants (PVs) of strains M6 and 7a1. All PVs produced larger colonies than those produced by the parental strains on NA (Figure 1). These results were in agreement with the fact that two assessed variants of each strain (M6V1 and M6V2 for strain M6, and 7a1V1 and 7a1V2 for strain 7a1) generated colonies that possessed a significantly higher number of cells than the corresponding parental strain colonies (Figure 2). All variant colonies also showed a translucent and fuzzy appearance in contrast to parental strain colonies that were opaque and smooth (shown for selected variants in Figure 1). In addition, SEM analyses showed that variant cells of both strains possessed a slightly increased roughness than parental type cells (Figure S1). These morphological alterations could be result of changes in surface-exposed moieties such as fimbriae/pili, lipopolysaccharide (LPS) and extracellular polysaccharides, as shown for other bacteria [8,11,41,42,47–49]. Although this question should be further investigated in future studies, here we show that under tested conditions, the characterized variants of strains M6 and 7a1 lacked the ability to produce type IV pili (T4P), which could contribute to these morphological alterations (Figure 6).

Consistent with the lack of ability of the characterized variants to produce T4P, no twitching motility haloes could be detected around colonies of all M6 and 7a1 PVs isolated in this study, and twitching zones could not be detected at the edge of colonies of PVs (Movies S3 and S4). T4P have been shown to be important virulence determinants of *A. citrulli* M6, with T4P mutants of this bacterium being significantly affected in virulence [29]. Therefore, the reduced virulence observed for the assessed M6 and 7a1 variants in seed transmission assays on melon and watermelon, respectively (Figure 5), could be due, at least partially, to their lack of ability to produce this type of pilus.

Additional phenotypic similarities among the characterized M6 variants (M6V1 and M6V2) and T4P mutants of strain M6 include colony morphological appearance, increased swimming motility and reduced biofilm formation ability [29]. Although polar flagella have been inferred as factors required for biofilm formation in several bacterial species [50,51], Bahar et al. [30,31] showed that under tested conditions, polar flagella do not appear to play a major role in biofilm formation of *A. citrulli*. In contrast, T4P were shown to significantly contribute to biofilm formation in *A. citrulli* M6 [29,30]. Therefore, the reduced biofilm formation of variants M6V1 and M6V2 could be explained by their lack of ability to produce T4P.

Given the results of biofilm assays with M6 variants, and the knowledge about the importance of T4P for biofilm formation in *A. citrulli* M6 [30], we expected that also 7a1 variants would show reduced biofilm formation ability. Surprisingly, however, despite lacking the abilities to produce both T4P and polar flagella, 7a1 variants had pronounced biofilm formation ability than the parental strain under tested conditions (Figure 4). As mentioned polar flagella were not shown to play a significant role in biofilm formation of *A. citrulli* M6 [31]. While T4P were shown to be important for attachment and biofilm formation in *A. citrulli* M6 and in other bacteria [52], in some cases, like in the plant pathogenic bacterium *Xylella fastidiosa*, T4P was shown to negatively affect biofilm formation. In this bacterium, mutants impaired in T4P production had pronounced biofilm formation ability than the wild type [53,54]. It is also known that nonfimbrial adhesins may be involved in surface attachment and biofilm formation [54,55], and that these features can also be influenced by the level of hydrophobicity of the cell surface [56,57]. Further investigation is necessary to understand the pronounced biofilm formation in 7a1 variants. Enhanced biofilm formation by these variants could confer increased survival under adverse conditions, such as environmental stresses or exposure to host defense mechanisms [58].

In contrast to M6 variants, 7a1 variants also lacked swimming motility ability (Figure S2) and we were not able to detect polar flagella in 7a1 variants by electron microscopy (Figure 6B). Loss of the ability to produce polar flagellum has been observed in PVs of other bacterial species including the closely related *Acidovorax* *radicis* (see below) [44]. Nevertheless, we cannot discard the possibility that in the case of 7a1 variants, this phenotype might not be directly associated with phenotypic variation, since loss of the ability to produce polar flagellum, independently of the occurrence of phenotypic variation, has been often observed in our lab in the case of group II strains of *A. citrulli* [26,31].

Although phenotypic variation can be reversible [11], no reversion of variants to parental strain was observed in our study, although multiple subcultures were done for the variants in the lab. Similar irreversible phase variants were already reported on various bacteria including *Burkholderia* *ambifaria* [43], *Acidovorax* *radicis* [44] and *Azospirillum* *lipoferum* [13]. For both M6 and 7a1, at least one of the variants displayed significant changes in AUC parameter of respiration kinetics, indicating that the mutational changes also effected carbon source utilization, i.e., central parts of the cellular energy metabolism (Figure S3).

Phenotypic variation is often associated with large DNA rearrangements and PVs can be distinguished from their parental strains by diverse DNA fingerprinting methods [13,39–41]. Here we showed that 7a1 PVs slightly differed from the parental strain in their PFGE profile following restriction with XbaI and variant 7a1V2 slightly differed from the parental strain (and from variant 7a1V1) in REP-PCR. In contrast, no differences could be found among the two M6 variants and parental strain M6 in any DNA fingerprinting method used in this study, suggesting that in this strain, phenotypic variation is associated with slight genetic changes or epigenetic alterations. As an example of slight genetic changes that are hard to detect by DNA fingerprinting and lead to phenotypic variation, in the closely related, non-pathogenic, plant root-associated bacterium *Acidovorax* *radicis* N35, irreversible phenotypic variation was shown to occurs at a frequency of ~10^-3^ [44]. *A* *. radicis* N35 variants showed a smooth colony type (in contrast to the rough type of the parental strain), lost their flagella, and had decreased competitive endophytic root colonization relative to the parental strain [44].

Irreversible phenotypic variation was also reported in the plant beneficial bacterium *Pseudomonas fluorescens* F113. In this bacterium, phenotypic variation is associated with mutations in the GacS/GacA two component regulatory system [42]. The GacS/GacA system is present in a wide variety of Gram-negative bacteria and has been studied manly in enteric bacteria and fluorescent pseudomonads. This system controls the production of secondary metabolites and extracellular enzymes involved in pathogenicity, biocontrol of soilborne phytopathogens, ecological fitness or stress tolerance [59,60]. Interestingly, some of the phenotypes detected for the PVs of *A. citrulli* M6 (eg, translucent appearance and increased swimming motility) are similar to those previously described for PVs of *P. fluorescens* F113 [15,42]. It has been shown that in *P. fluorescens*, the GacS/GacA system regulates swimming motility by a repression pathway, which explains the increased swimming motility of the PVs relative to the parental strain [15]. While no genes have been annotated as *gacA* or *gacS* in *A. citrulli* AAC00-1, the AAC00-1 genome has genes encoding products with 34 to 38% identity with *P. fluorescens* GacA (the response regulator of the GacS/GacA system). Comparative genomics between selected variants and parental strain in combination with mutagenesis/overexpression of selected genes in the parental strain will provide insights into genetic/epigenetic elements regulating phenotypic variation in *A. citrulli*.

In *P. fluorescens*, genetic rearrangements associated with phenotypic variation were found to be mediated by two site-specific recombinases, XerD and Sss [14]. *A. citrulli* possesses a high number of genes that could mediate DNA rearrangements and mutations, including 59 genes encoding proteins with integrase catalytic subunits, 45 transposases/transposases family proteins and 3 recombinases (according to the annotation of strain AAC00-1). Interestingly, the AAC00-1 genome possesses two genes with high similarity to *P. fluorescens* XerD and Sss (Aave_1313, annotated as tyrosine recombinase XerD, 72% similarity; and Aave_0801, annotated as phage integrase family protein, 59% similarity, respectively; gene names according to the AAC00-1 annotation). In the future we plan to assess whether these genes or other recombinases/transposases are involved in phenotypic variation of *A. citrulli*.

We have observed that variants of the same strain are highly similar or identical in many physiological activities. Nevertheless, further characterization of selected variants revealed some phenotypic differences between variants of the same strain. For example, variant M6V2 showed significantly higher swimming motility than variant M6V1 (Figure 3). Also, variant 7a1V2 had significantly (in two out of three experiments) more CFUs per colony (Figure 2) and showed significantly higher biofilm formation ability (Figure 4) than variant 7a1V1. These variants also showed some differences in their colony morphology (Figure 1) and a slight difference in REP-PCR (Figure 7). Moreover, variant 7a1V2 cells were slightly but significantly smaller in their size than 7a1V1 and parental type cells (not shown). Some differences between variants of the same strain were also found in their Omnilog metabolic profiles. Altogether, these findings suggest that phenotypic variation in a given *A. citrulli* strain may be associated with different genetic or epigenetic alterations. As mentioned above, comparative genomic analysis between *A. citrulli* parental strains and variants may provide clues into regulation of phenotypic variation in this bacterium.

It is important to stress that while all PVs isolated in this study shared similar characteristics in terms of colony morphology appearance, colony size and lack of formation of T4P-mediated twitching haloes, only two PVs of each strain were deeply characterized in terms of virulence, biofilm formation and swimming motility. Due to the demonstrated effects of T4P in these features in *A. citrulli* [29], and the fact that all isolated variants lacked T4P-mediated twitching motility, it is reasonable to suggest that the PVs characterized in this study are highly representative; however this question should be carefully evaluated in the future.

In this study, PVs of both strains could be obtained following isolation of the bacteria from infected plants. Intriguingly, all tested PVs had reduced virulence than their parental strains at tested conditions. The understanding of the implication of this finding for *A. citrulli* fitness and for the interaction of *A. citrulli* with its host plants requires further investigation. Could reduced virulence, under certain circumstances, be considered as an advantage for the pathogen? Alternatively, could reduced virulence be associated with some other (unknown at the moment) beneficial trait for the bacterium, in the same or in a different environmental niche? For instance, PVs of *Burkholderia* *ambifaria* - a pathogen that can cause severe respiratory infections in people suffering from cystic fibrosis- were shown to be less virulent than the wild type in four different animal virulence models. However, *B* *. ambifaria* is also a plant growth-promoting bacterium and plant inoculation with the above PVs revealed an increased ability to colonize the rhizosphere. This may suggest that in this bacterium, phenotypic variation has a role in niche adaptation [43]. Interestingly, association of reduced virulence with phenotypic variation was also reported in plant pathogenic *Ralstonia solanacearum* [61–63] and *Xanthomonas* *oryzae* pv. *oryzae* [64]. In both bacterial species, PVs were also shown to be deficient in production of extracellular polysaccharides (EPS) [63,64], and in the case of *R. solanacearum*, PVs also showed increased motility, as similar as we observed in PVs of *A. citrulli* M6 [61]. Exploration of the mechanism and effects of phenotypic variation in *A. citrulli* may provide insights into fitness strategies and pathogenicity of this bacterium, which are highly needed to develop more efficient tools to cope with BFB.

## Supporting Information

Appendix S1 **Supplemental file describing details of the statistical analyses.**
(PDF)

Figure S1 **Scanning electron micrographs of cells from NA-grown colonies of *A. citrulli* M6 parental strain and variants.** Fourty-eight-h-old colonies were excised from NA plates, fixed with glutaraldehyde, gradually dehydrated in ethanol, dried in a Critical Point Dryer, and finally coated with gold particles. Pictures were taken at a magnification level of 20,000X. **A**, parental strain M6; **B**, variant M6V1; **C**, variant M6V2.
(TIFF)

Figure S2 **Images showing absence of swimming motility in PVs of *A. citrulli* 7a1.** **A**, parental strain 7a1; **B**, variant 7a1V1; **C**, variant 7a1V2. Strains were inoculated onto the center of soft agar NA plates, and pictures were taken after 24 h of incubation at 28^°^C. Note the absence of swimming zones in the variants in contrast to the parental strain.
(TIFF)

Figure S3 **Dynamics of tetrazolium reduction in selected Biolog GEN III MicroPlate wells.** Six wells showing distinguished patterns between parental strains and variants are shown for M6 (**A**) and 7a1 (**B**). For each strain, the parental strain (M6, 7a1) is colored black, variant 1 (M6V1, 7a1V1) is colored red, and variant 2 (M6V2, 7a1V2) is colored blue. Results are shown from three independent experiments, with two replicates per strain, yielding altogether six curves per strain. Details on statistical differences between parental strains and variants are shown in Appendix S1.
(TIFF)

Movie S1 **Time lapse movie focusing on the edge of a colony of parental strain M6 after growth of 72 h on NA.** Images were captured every 15 min for a period of 6 h using a digital camera coupled to a light microscope, and time lapse movies were generated with ImageJ software.
(ZIP)

Movie S2 **Time lapse movie focusing on the edge of a colony of parental strain 7a1 after growth of 72 h on NA.** Images were captured every 15 min for a period of 6 h using a digital camera coupled to a light microscope, and time lapse movies were generated with ImageJ software.
(ZIP)

Movie S3 **Time lapse movie focusing on the edge of a colony of phenotypic variant M6V1 after growth of 72 h on NA.** Images were captured every 15 min for a period of 6 h using a digital camera coupled to a light microscope, and time lapse movies were generated with ImageJ software.
(ZIP)

Movie S4 **Time lapse movie focusing on the edge of a colony of phenotypic variant 7a1V1 after growth of 72 h on NA.** Images were captured every 15 min for a period of 6 h using a digital camera coupled to a light microscope, and time lapse movies were generated with ImageJ software.
(ZIP)

Movie S5 **Time lapse movie focusing on the edge of a colony of the M6 *pilM*^-^ mutant (M6-2-23) after growth of 72 h on NA.** Images were captured every 15 min for a period of 6 h using a digital camera coupled to a light microscope, and time lapse movies were generated with ImageJ software.
(ZIP)
