# Supplementary material for: Phenotypic Variation in the Plant Pathogenic Bacterium Acidovorax citrulli
Source: PLoS One. 2013 Sep 2;8(9):e73189. doi: 10.1371/journal.pone.0073189 (PMC3759439; doi:10.1371/journal.pone.0073189)
Supplement: Appendix S1 — Supplemental file describing details of the statistical analyses. (PDF) [file pone.0073189.s001.pdf]

Appendix S1
to
"Phenotypic variation in the plant pathogenic bacterium *Acidovorax citrulli*"
-
Details of the statistic analysis

Ram Kumar Shrestha
The Hebrew University of Jerusalem,
Rehovot 76100, Israel

Tally Rosenberg
The Hebrew University of Jerusalem,
Rehovot 76100, Israel

Daria Makarovsky
The Hebrew University
of Jerusalem,
Rehovot 76100, Israel

Noam Eckshtein-Levi
The Hebrew University
of Jerusalem,
Rehovot 76100, Israel

Einat Zelinger
The Hebrew University
of Jerusalem,
Rehovot 76100, Israel

June Kopelowitz
Savyon Diagnostics Ltd.,
Ashdod, Israel

Johannes Sikorski
Leibniz Institute DSMZ,
Braunschweig, Germany

Saul Burdman
The Hebrew University
of Jerusalem,
Rehovot 76100, Israel

Abstract

This file includes all statistical details to the manuscript "Phenotypic variation in the plant pathogenic bacterium *Acidovorax citrulli*".

This is a **Sweave** file. **Sweave** is a tool that allows to embed the R code for complete data analyses in LaTeX documents. The purpose is to create dynamic reports, which can be updated automatically if data or analysis change. Instead of inserting a prefabricated graph or table into the report, the master document contains the R code necessary to obtain it. When run through R, all data analysis output (tables, graphs, etc.) is created on the fly and inserted into a final LaTeX document. The report can be automatically updated if data or analysis change, which allows for truly reproducible research.

Hence, this file informs in detail which statistical analysis have been performed and documents the way this has been achieved.

Keywords: *Acidovorax citrulli*, CFU, biofilm formation, swimming motility, carbon substrate respiration kinetics.

Details on **Sweave** are dokumented elsewhere(Leisch 2002). R code is indicated by an R> prompt:

```
R> here comes some R code
```

Load the R packages that are necessary for the analysis.

```
R> # general
R> rm(list=ls(all=TRUE))   # removes all functions, libraries, data etc
R> # load packages
R> library(opm)
R> library(ggplot2)
R> library(multcomp)
R> library(sandwich)
```

Load the data.

```
R> biofilm <- read.table('biofilm.txt', header = TRUE, sep = "\t", dec = ",")
R> cfu <- read.table('CFU.txt', header = TRUE, sep = "\t", dec = ",")
R> swimming <- read.table('swimmingM6.txt', header = TRUE, sep = "\t", dec = ",")
```

1. Analysis of CFU per colony

1.1. Structure and summary of CFU data

Display the first six lines of the data set.

```
R> head(cfu)

  experiment strain variants Treatment No.of.Colony CFU.per.colony
1      first     M6 parental        M6            1      144000000
2      first     M6 parental        M6            2      194000000
3      first     M6 parental        M6            3      152000000
4      first     M6 parental        M6            4      256000000
5      first     M6 parental        M6            5      228000000
6      first     M6       V1      M6V1            1      482000000
```

Display summary statistics of each column.

```
R> summary(cfu)

  experiment strain       variants  Treatment   No.of.Colony
 first :30   7a1:45   parental:30   7a1   :15   Min.   :1
 second:30   M6 :45   V1      :30   7a1V1:15   1st Qu.:2
 third :30            V2      :30   7a1V2:15   Median :3
                                    M6    :15   Mean   :3
                                    M6V1  :15   3rd Qu.:4
                                    M6V2  :15   Max.   :5
 CFU.per.colony
```

```
 Min.   :6.800e+07
 1st Qu.:2.390e+08
 Median :5.790e+08
 Mean   :5.971e+08
 3rd Qu.:7.780e+08
 Max.   :1.580e+09
```

1.2. Figure CFU

Below is the code in order to reproduce Figure 1.

```
R> cfu$strain <- factor(cfu$strain, levels=c("M6", "7a1"),ordered=TRUE)
R> cfu$strain.experiment <- paste(cfu$strain, " (",
                                  cfu$experiment, ")", sep = "")
R> cfu$strain.experiment <- factor(cfu$strain.experiment,
                                      levels=c("M6 (first)","7a1 (first)",
                                               "M6 (second)","7a1 (second)",
                                               "M6 (third)", "7a1 (third)"),
                                      ordered=TRUE)
R> ggplot(cfu, aes(factor(variants), CFU.per.colony)) +
     geom_boxplot(outlier.colour = "grey", outlier.size = 0.001) +
     geom_jitter(size = 2, position=position_jitter(width=0.3)) +
     theme_bw() +
     xlab("\nstrains") +
     ylab("cfu/colony \n") +
     facet_wrap( ~ strain.experiment, nrow = 3) +
     scale_y_log10(breaks=c(100000000,200000000,300000000,500000000,
                           1000000000,1500000000),
                   limits=c(100000000,1700000000)) +
     theme(strip.text.x = element_text(size = 14)) +
     theme(axis.text = element_text(size = 12)) +
     theme(axis.title = element_text(size = 14))
```

1.3. Statistics M6 CFU: output of significance tests

Define subsets for the three replicate experiments

```
R> firstM6 <- subset(cfu, strain.experiment == "M6 (first)")
R> secondM6 <- subset(cfu, strain.experiment == "M6 (second)")
R> thirdM6 <- subset(cfu, strain.experiment == "M6 (third)")
```

Apply **multcomp** statistics (Hothorn, Bretz, and Westfall 2008; Herberich, Sikorski, and Hothorn 2010) to the ***first*** replicate on CFU analysis of strain M6 and its variants. The stars indicate the level of statistical significance between pairwise comparison of groups.

```
R> amod <- aov(CFU.per.colony ~ Treatment, data = firstM6)
R> cfu.firstM6 <- glht(amod, mcp(Treatment = "Tukey"), vcov = vcovHC)
```

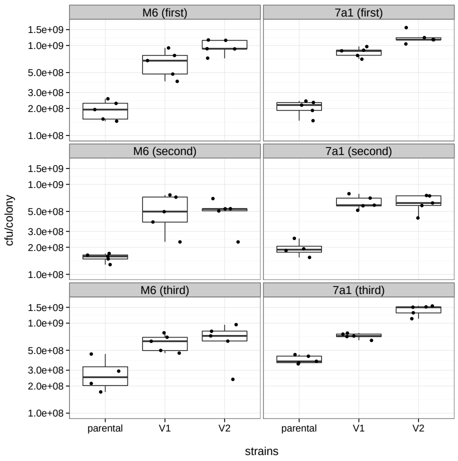

Figure 1: **CFU/colony was determined following serial dilution plating of five colonies per strain.** The top, middle, and bottom rows show the results of the first, second, and third independent replicate, respectively. Each dot indicates the CFU of a single colony. The box-and-whisker plots indicate minimum, first quartile, median, third quartile, and maximum values.

Below, the results of this statistical analyses are shown as the output from the **multcomp** calculations.

```
R> summary(cfu.firstM6)

         Simultaneous Tests for General Linear Hypotheses

Multiple Comparisons of Means: Tukey Contrasts


Fit: aov(formula = CFU.per.colony ~ Treatment, data = firstM6)

Linear Hypotheses:
                  Estimate Std. Error t value Pr(>|t|)
M6V1 - M6 == 0   460400000  111973658   4.112  0.00368 **
M6V2 - M6 == 0   776000000   93378799   8.310  < 0.001 ***
M6V2 - M6V1 == 0 315600000  141774822   2.226  0.10213
---
Signif. codes:  0 '***' 0.001 '**' 0.01 '*' 0.05 '.' 0.1 ' ' 1
(Adjusted p values reported -- single-step method)
```

The last column `Pr(>|t|)` gives the so called probability values of falsely rejecting a statistical hypothesis (p-value). The hypothesis is that there is no difference between the groups that are compared pairwise to each other. The data are from the ***first*** replicate on CFU analysis of strain M6 and its variants (see upper left panel in Figure 1). The output above reads as follows.

- Strains M6V1 and M6 are significantly different at p = 0.01
- Strains M6V2 and M6 are significantly different at p = 0.001
- Strains M6V1 and M6V2 are **not** significantly different at p = 0.05

All other **multcomp** analyses follow the same principle.

Apply **multcomp** statistics (Hothorn *et al.* 2008; Herberich *et al.* 2010) for the ***second*** replicate on CFU analysis of strain M6 and its variants. The stars indicate the level of statistical significance between pairwise comparison of groups.

```
R> amod <- aov(CFU.per.colony ~ Treatment, data = secondM6)
R> cfu.secondM6 <- glht(amod, mcp(Treatment = "Tukey"), vcov = vcovHC)
R> summary(cfu.secondM6)
```

```
         Simultaneous Tests for General Linear Hypotheses

Multiple Comparisons of Means: Tukey Contrasts


Fit: aov(formula = CFU.per.colony ~ Treatment, data = secondM6)

Linear Hypotheses:
                  Estimate Std. Error t value Pr(>|t|)
M6V1 - M6 == 0   364400000  112819325   3.230  0.01701 *
M6V2 - M6 == 0   345200000   84471889   4.087  0.00377 **
M6V2 - M6V1 == 0 -19200000  140457467  -0.137  0.98918
---
Signif. codes:  0 '***' 0.001 '**' 0.01 '*' 0.05 '.' 0.1 ' ' 1
(Adjusted p values reported -- single-step method)
```

Apply **multcomp** statistics (Hothorn *et al.* 2008; Herberich *et al.* 2010) for the ***third*** replicate on CFU analysis of strain M6 and its variants. The stars indicate the level of statistical significance between pairwise comparison of groups.

```
R> amod <- aov(CFU.per.colony ~ Treatment, data = thirdM6)
R> cfu.thirdM6 <- glht(amod, mcp(Treatment = "Tukey"), vcov = vcovHC)
R> summary(cfu.thirdM6)
```

```
         Simultaneous Tests for General Linear Hypotheses

Multiple Comparisons of Means: Tukey Contrasts


Fit: aov(formula = CFU.per.colony ~ Treatment, data = thirdM6)

Linear Hypotheses:
                  Estimate Std. Error t value Pr(>|t|)
M6V1 - M6 == 0   373200000   98495685   3.789  0.00651 **
M6V2 - M6 == 0   433600000  154020129   2.815  0.03716 *
M6V2 - M6V1 == 0  60400000  151572425   0.398  0.91499
---
Signif. codes:  0 '***' 0.001 '**' 0.01 '*' 0.05 '.' 0.1 ' ' 1
(Adjusted p values reported -- single-step method)
```

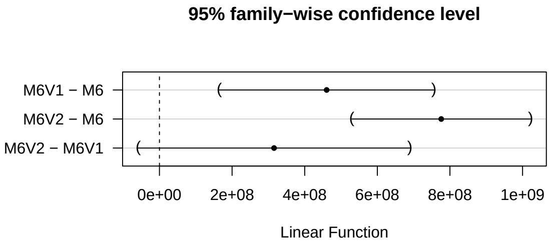

Figure 2: Plot of the statistical comparison of the first replicate on CFU analysis of strain M6 and its variants. The filled black circle indicates the point estimator of difference between the mean of groups. 95% confidence intervals are indicated by horizontal bars and parentheses. In pairwise comparisons, if the 95% confidence interval includes zero (dashed vertical line) there is no significant difference between the group means. Conversely, if zero is not included, a significant difference is indicated. Furthermore, the more distant the 95% confidence interval is from zero, the larger the biological effect size, i.e. the real difference between the groups. Thus, beyond the number of stars indicating the level of statistical significance (Signif. codes: 0 '***' 0.001 '**' 0.01 '*' 0.05 '.' 0.1 ' ' 1), the 95% confidence interval is of large relevance for the biological interpretation of the statistical comparison.

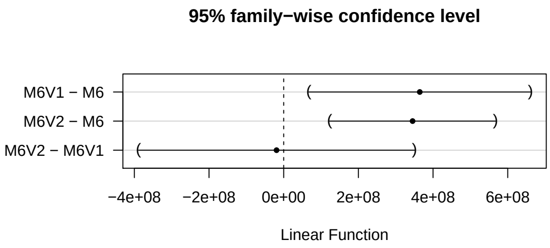

Figure 3: Plot of the statistical comparison of the second replicate on CFU analysis of strain M6 and its variants. For interpretation of the plot, see legend of Figure 2.

1.4. Statistics M6 CFU: plot of significance tests

The results from the statistical analyses above can be plotted graphically. The interpretation of these graphs is explained in the legend of the figure. All other graphical **multcomp** outputs follow the same principle. Below is the `R` code for Figure 2.

```
R> par(mai = c(1,2.0,1,0.5))
R> plot(cfu.firstM6)
```

Below is the `R` code for Figure 3.

```
R> par(mai = c(1,2.0,1,0.5))
R> plot(cfu.secondM6)
```

Below is the `R` code for Figure 4.

```
R> par(mai = c(1,2.0,1,0.5))
R> plot(cfu.thirdM6)
```

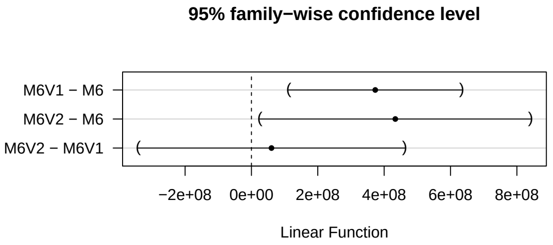

Figure 4: Plot of the statistical comparison of the third replicate on CFU analysis of strain M6 and its variants. For interpretation of the plot, see legend of Figure 2.

1.5. Statistics 7a1 CFU: output of significance tests

Define subsets for the three replicate experiments

```
R> first7a1 <- subset(cfu, strain.experiment == "7a1 (first)")
R> second7a1 <- subset(cfu, strain.experiment == "7a1 (second)")
R> third7a1 <- subset(cfu, strain.experiment == "7a1 (third)")
```

Apply **multcomp** statistics (Hothorn *et al.* 2008; Herberich *et al.* 2010) to the ***first*** replicate on CFU analysis of strain 7a1 and its variants. The stars indicate the level of statistical significance between pairwise comparison of groups.

```
R> amod <- aov(CFU.per.colony ~ Treatment, data = first7a1)
R> cfu.first7a1 <- glht(amod, mcp(Treatment = "Tukey"), vcov = vcovHC)
R> summary(cfu.first7a1)

         Simultaneous Tests for General Linear Hypotheses

Multiple Comparisons of Means: Tukey Contrasts


Fit: aov(formula = CFU.per.colony ~ Treatment, data = first7a1)

Linear Hypotheses:
                    Estimate Std. Error t value Pr(>|t|)
7a1V1 - 7a1 == 0   6.384e+08  5.526e+07  11.553   <0.001 ***
7a1V2 - 7a1 == 0   1.026e+09  1.044e+08   9.830   <0.001 ***
7a1V2 - 7a1V1 == 0 3.880e+08  1.149e+08   3.376   0.0127 *
---
Signif. codes:  0 '***' 0.001 '**' 0.01 '*' 0.05 '.' 0.1 ' ' 1
(Adjusted p values reported -- single-step method)
```

Apply **multcomp** statistics (Hothorn *et al.* 2008; Herberich *et al.* 2010) to the ***second*** replicate on CFU analysis of strain 7a1 and its variants. The stars indicate the level of statistical significance between pairwise comparison of groups.

```
R> amod <- aov(CFU.per.colony ~ Treatment, data = second7a1)
R> cfu.second7a1 <- glht(amod, mcp(Treatment = "Tukey"), vcov = vcovHC)
R> summary(cfu.second7a1)
```

```
         Simultaneous Tests for General Linear Hypotheses

Multiple Comparisons of Means: Tukey Contrasts


Fit: aov(formula = CFU.per.colony ~ Treatment, data = second7a1)

Linear Hypotheses:
                    Estimate Std. Error t value Pr(>|t|)
7a1V1 - 7a1 == 0   458000000   61493089   7.448   <1e-04 ***
7a1V2 - 7a1 == 0   449600000   73598234   6.109   <1e-04 ***
7a1V2 - 7a1V1 == 0  -8400000   86791129  -0.097    0.995
---
Signif. codes:  0 '***' 0.001 '**' 0.01 '*' 0.05 '.' 0.1 ' ' 1
(Adjusted p values reported -- single-step method)
```

Apply **multcomp** statistics (Hothorn *et al.* 2008; Herberich *et al.* 2010) to the ***third*** replicate on CFU analysis of strain 7a1 and its variants. The stars indicate the level of statistical significance between pairwise comparison of groups.

```
R> amod <- aov(CFU.per.colony ~ Treatment, data = third7a1)
R> cfu.third7a1 <- glht(amod, mcp(Treatment = "Tukey"), vcov = vcovHC)
R> summary(cfu.third7a1)
```

```
         Simultaneous Tests for General Linear Hypotheses

Multiple Comparisons of Means: Tukey Contrasts


Fit: aov(formula = CFU.per.colony ~ Treatment, data = third7a1)

Linear Hypotheses:
                    Estimate Std. Error t value Pr(>|t|)
7a1V1 - 7a1 == 0   3.272e+08  3.288e+07   9.950  < 1e-05 ***
7a1V2 - 7a1 == 0   1.006e+09  9.534e+07  10.548  < 1e-05 ***
7a1V2 - 7a1V1 == 0 6.784e+08  9.639e+07   7.038 1.24e-05 ***
---
Signif. codes:  0 '***' 0.001 '**' 0.01 '*' 0.05 '.' 0.1 ' ' 1
(Adjusted p values reported -- single-step method)
```

1.6. Statistics 7a1 CFU: plot of significance tests

Below is the R code for Figure 5.

```
R> par(mai = c(1,2.0,1,0.5))
R> plot(cfu.first7a1, xlim = c(-10,1300000000))
```

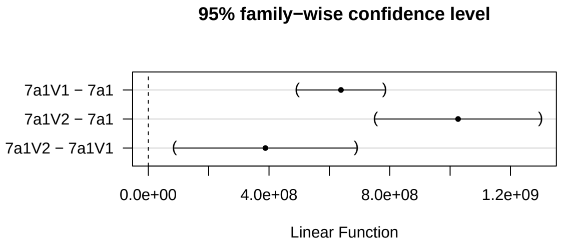

Figure 5: Plot of the statistical comparison of the first replicate on CFU analysis of strain 7a1 and its variants. For interpretation of the plot, see legend of Figure 2.

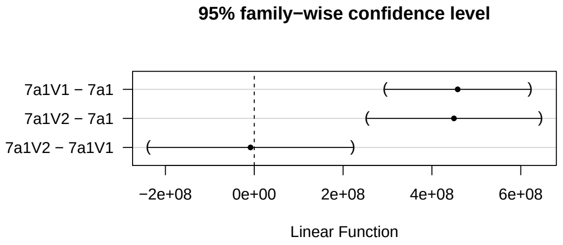

Figure 6: Plot of the statistical comparison of the second replicate on CFU analysis of strain 7a1 and its variants. For interpretation of the plot, see legend of Figure 2.

Below is the R code for Figure 6.

```
R> par(mai = c(1,2.0,1,0.5))
R> plot(cfu.second7a1)
```

Below is the R code for Figure 7.

```
R> par(mai = c(1,2.0,1,0.5))
R> plot(cfu.third7a1, xlim = c(-10,1300000000))
```

2. Analysis of swimming motility

2.1. Structure and summary of swimming motility data

Display the first six lines of the data set.

```
R> head(swimming)
```

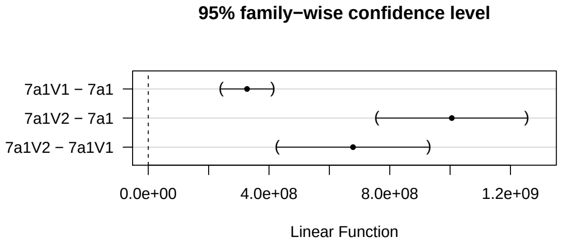

Figure 7: Plot of the statistical comparison of the third replicate on CFU analysis of strain 7a1 and its variants. For interpretation of the plot, see legend of Figure 2.

```
  experiment variants Treatment Replicate Swimming.area..mm2.
1      first       M6        M6         1              653.99
2      first       M6        M6         2              466.44
3      first       M6        M6         3              370.02
4      first       M6        M6         4              398.30
5      first       M6        M6         5              603.36
6      first       V1     M6-V1         1             1639.47
```

Display summary statistics of each column.

```
R> summary(swimming)
```

```
  experiment variants Treatment    Replicate     Swimming.area..mm2.
 first :15   M6:13    M6   :13   Min.   :1.000   Min.   : 370.0
 second:24   V1:13    M6-V1:13   1st Qu.:2.000   1st Qu.: 655.2
             V2:13    M6-V2:13   Median :4.000   Median :1057.9
                                 Mean   :3.923   Mean   :1268.7
                                 3rd Qu.:5.000   3rd Qu.:1898.5
                                 Max.   :8.000   Max.   :3325.3
```

2.2. Figure swimming motility

Below is the code in order to reproduce Figure 8.

```
R> ggplot(swimming, aes(factor(variants), Swimming.area..mm2.)) +
     geom_boxplot(outlier.colour = "grey", outlier.size = 0.001) +
     geom_jitter(aes(shape = experiment),
                 position=position_jitter(width=0.3), size = 4) +
     theme_bw() +
     scale_shape_manual(values = c(17,2)) +
     xlab("\n strains") +
     ylab("swimming area [mm^2] \n") +
     theme(strip.text.x = element_text(size = 20)) +
     theme(axis.text = element_text(size = 16)) +
     theme(axis.title = element_text(size = 18)) +
     theme(legend.position = c(.8, .8)) +
     scale_x_discrete(breaks=c("M6", "V1", "V2"),
                      labels=c("M6", "M6V1", "M6V2"))
```

Apply **multcomp** statistics (Hothorn *et al.* 2008; Herberich *et al.* 2010) to the ***first*** data on swimming motility of strain M6 and its variants. The stars indicate the level of statistical significance between pairwise comparison of groups.

```
R> amod <- aov(Swimming.area..mm2. ~ Treatment, data = swimming)
R> swimming.xM6 <- glht(amod, mcp(Treatment = "Tukey"), vcov = vcovHC)
R> summary(swimming.xM6)
```

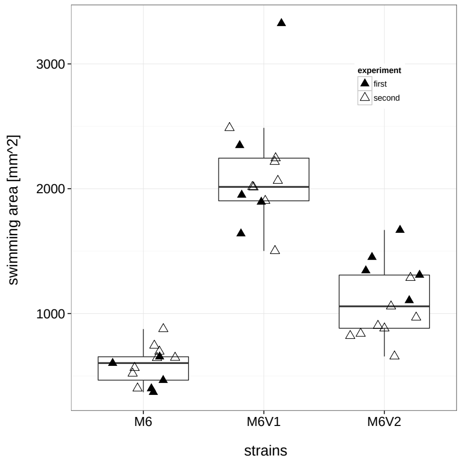

Figure 8: **Phenotypic variants M6V1 and M6V2 possess increased swimming motility than the parental strain.** Swimming assays were performed on soft NA plates (0.3 % agar). Swimming areas were measured after 24 hours of incubation at 28 degree C. The data of the first and the second replicate experiment are shown. Each triangle indicates an individual experiment. The box-and-whisker plot, indicating minimum, first quartile, median, third quartile, and maximum values, summarize both the first and the second replicate.

```
         Simultaneous Tests for General Linear Hypotheses

Multiple Comparisons of Means: Tukey Contrasts


Fit: aov(formula = Swimming.area..mm2. ~ Treatment, data = swimming)

Linear Hypotheses:
                   Estimate Std. Error t value Pr(>|t|)
M6-V1 - M6 == 0     1539.65     136.92  11.245   <1e-05 ***
M6-V2 - M6 == 0      516.13      95.09   5.428   <1e-05 ***
M6-V2 - M6-V1 == 0 -1023.52     155.11  -6.599   <1e-05 ***
---
Signif. codes:  0 '***' 0.001 '**' 0.01 '*' 0.05 '.' 0.1 ' ' 1
(Adjusted p values reported -- single-step method)
```

2.3. Statistics Swimming motility: plot of significance tests

Below is the `R` code for Figure 9.

```
R> par(mai = c(1,2.0,1,0.5))
R> plot(swimming.xM6)
```

3. Analysis of biofilm

3.1. Structure and summary of biofilm data

Display the first six lines of the data set.

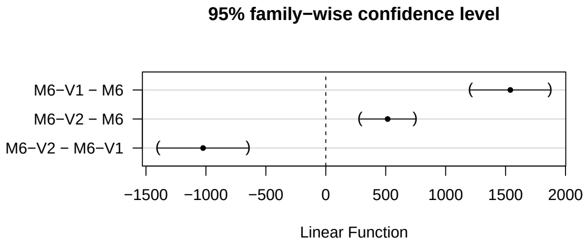

Figure 9: Plot of the statistical comparison of the swimming motility analysis of strain M6 and its variants. For interpretation of the plot, see legend of Figure 2.

```
R> head(biofilm)

  experiment strain variants Treatment Replicate OD595nm
1      first     M6 parental        M6         1  0.2630
2      first     M6 parental        M6         2  0.1534
3      first     M6 parental        M6         3  0.1273
4      first     M6 parental        M6         4  0.1188
5      first     M6 parental        M6         5  0.1145
6      first     M6       V1      M6V1         1  0.1119
```

Display summary statistics of each column.

```
R> summary(biofilm)

  experiment strain       variants  Treatment    Replicate      OD595nm
 first :30   7a1:75   parental:50   7a1   :25   Min.   : 1   Min.   :0.0338
 second:60   M6 :75   V1      :50   7a1V1:25    1st Qu.: 3   1st Qu.:0.1551
 third :60            V2      :50   7a1V2:25    Median : 5   Median :0.2092
                                    M6    :25   Mean   : 5   Mean   :0.3179
                                    M6V1  :25   3rd Qu.: 7   3rd Qu.:0.3772
                                    M6V2  :25   Max.   :10   Max.   :1.7512
```

3.2. Figure biofilm

Below is the code in order to reproduce Figure 10.

```
R> biofilm$strain.experiment <- paste(biofilm$strain, " (",
                                      biofilm$experiment, ")", sep = "")
R> biofilm$strain.experiment <- factor(biofilm$strain.experiment,
                                       levels=c("M6 (first)","7a1 (first)",
                                                "M6 (second)","7a1 (second)",
                                                "M6 (third)", "7a1 (third)"),
                                       ordered=TRUE)
```

3.3. Statistics M6 biofilm: output of significance tests

Define subsets for the three replicate experiments

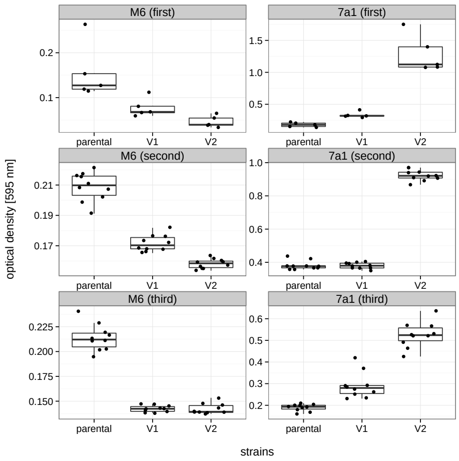

Figure 10: **Biofilm formation ability of strains M6 and 7a1 and their corresponding variants.** Cultures were grown in XVM2 medium for 48 hours in polystyrene ELISA plates at 28 degree C. Formed biofilms at the medium/air interface were stained with 0.01% crystal violet stain. Stained biofilms were resuspended with ethanol and quantitatively estimated by optical density measurements at 595 nm. The top, middle, and bottom rows show the results of the first, second, and third independent replicate experiment, respectively (with five replicates per strain in the first experiment and ten replicates per strain in second and third experiments). The box-and-whisker plots indicate minimum, first quartile, median, third quartile, and maximum values. Different letters indicate statistically significant differences ($p < 0.05$) among strains in each experiment.

```
R> firstM6 <- subset(biofilm, strain.experiment == "M6 (first)")
R> secondM6 <- subset(biofilm, strain.experiment == "M6 (second)")
R> thirdM6 <- subset(biofilm, strain.experiment == "M6 (third)")
```

Apply **multcomp** statistics (Hothorn *et al.* 2008; Herberich *et al.* 2010) to the ***first*** replicate on biofilm analysis of strain M6 and its variants. The stars indicate the level of statistical significance between pairwise comparison of groups.

```
R> amod <- aov(OD595nm ~ Treatment, data = firstM6)
R> biofilm.firstM6 <- glht(amod, mcp(Treatment = "Tukey"), vcov = vcovHC)
R> summary(biofilm.firstM6)

         Simultaneous Tests for General Linear Hypotheses

Multiple Comparisons of Means: Tukey Contrasts


Fit: aov(formula = OD595nm ~ Treatment, data = firstM6)

Linear Hypotheses:
                 Estimate Std. Error t value Pr(>|t|)
M6V1 - M6 == 0   -0.07808    0.03271  -2.387   0.0744 .
M6V2 - M6 == 0   -0.10912    0.03168  -3.445   0.0113 *
M6V2 - M6V1 == 0 -0.03104    0.01227  -2.531   0.0578 .
---
Signif. codes:  0 '***' 0.001 '**' 0.01 '*' 0.05 '.' 0.1 ' ' 1
(Adjusted p values reported -- single-step method)
```

Apply **multcomp** statistics (Hothorn *et al.* 2008; Herberich *et al.* 2010) to the ***second*** replicate on biofilm analysis of strain M6 and its variants. The stars indicate the level of statistical significance between pairwise comparison of groups.

```
R> amod <- aov(OD595nm ~ Treatment, data = secondM6)
R> biofilm.secondM6 <- glht(amod, mcp(Treatment = "Tukey"), vcov = vcovHC)
R> summary(biofilm.secondM6)

         Simultaneous Tests for General Linear Hypotheses

Multiple Comparisons of Means: Tukey Contrasts


Fit: aov(formula = OD595nm ~ Treatment, data = secondM6)

Linear Hypotheses:
                  Estimate Std. Error t value Pr(>|t|)
M6V1 - M6 == 0   -0.037330   0.003590 -10.398  < 1e-06 ***
M6V2 - M6 == 0   -0.050660   0.003299 -15.356  < 1e-06 ***
```

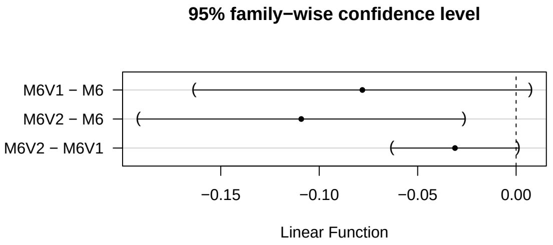

Figure 11: Plot of the statistical comparison of the first replicate on biofilm analysis of strain M6 and its variants. For interpretation of the plot, see legend of Figure 2.

```
M6V2 - M6V1 == 0 -0.013330   0.002062  -6.464 1.62e-06 ***
---
Signif. codes:  0 '***' 0.001 '**' 0.01 '*' 0.05 '.' 0.1 ' ' 1
(Adjusted p values reported -- single-step method)
```

Apply **multcomp** statistics (Hothorn *et al.* 2008; Herberich *et al.* 2010) to the ***third*** replicate on biofilm analysis of strain M6 and its variants. The stars indicate the level of statistical significance between pairwise comparison of groups.

```
R> amod <- aov(OD595nm ~ Treatment, data = thirdM6)
R> biofilm.thirdM6 <- glht(amod, mcp(Treatment = "Tukey"), vcov = vcovHC)
R> summary(biofilm.thirdM6)

         Simultaneous Tests for General Linear Hypotheses

Multiple Comparisons of Means: Tukey Contrasts


Fit: aov(formula = OD595nm ~ Treatment, data = thirdM6)

Linear Hypotheses:
                  Estimate Std. Error t value Pr(>|t|)
M6V1 - M6 == 0   -0.071690   0.004632 -15.478   <1e-07 ***
M6V2 - M6 == 0   -0.071720   0.004841 -14.816   <1e-07 ***
M6V2 - M6V1 == 0 -0.000030   0.002067  -0.015        1
---
Signif. codes:  0 '***' 0.001 '**' 0.01 '*' 0.05 '.' 0.1 ' ' 1
(Adjusted p values reported -- single-step method)
```

3.4. Statistics M6 biofilm: plot of significance tests

Below is the R code for Figure 11.

```
R> par(mai = c(1,2.0,1,0.5))
R> plot(biofilm.firstM6)
```

Below is the R code for Figure 12.

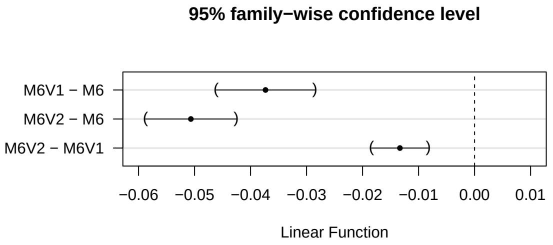

Figure 12: Plot of the statistical comparison of the second replicate on biofilm analysis of strain M6 and its variants. For interpretation of the plot, see legend of Figure 2.

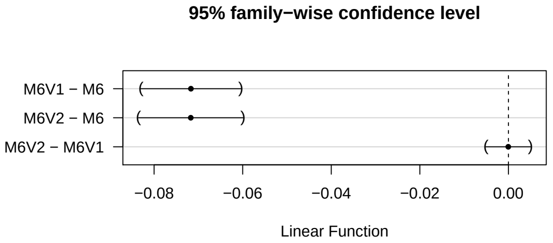

Figure 13: Plot of the statistical comparison of the third replicate on biofilm analysis of strain M6 and its variants. For interpretation of the plot, see legend of Figure 2.

```
R> par(mai = c(1,2.0,1,0.5))
R> plot(biofilm.secondM6, xlim = c(-0.06,0.01))
```

Below is the R code for Figure 13.

```
R> par(mai = c(1,2.0,1,0.5))
R> plot(biofilm.thirdM6)
```

3.5. Statistics 7a1 biofilm: output of significance tests

Define subsets for the three replicate experiments

```
R> first7a1 <- subset(biofilm, strain.experiment == "7a1 (first)")
R> second7a1 <- subset(biofilm, strain.experiment == "7a1 (second)")
R> third7a1 <- subset(biofilm, strain.experiment == "7a1 (third)")
```

Apply **multcomp** statistics (Hothorn *et al.* 2008; Herberich *et al.* 2010) to the ***first*** replicate on biofilm analysis of strain 7a1 and its variants. The stars indicate the level of statistical significance between pairwise comparison of groups.

```
R> amod <- aov(OD595nm ~ Treatment, data = first7a1)
R> biofilm.first7a1 <- glht(amod, mcp(Treatment = "Tukey"), vcov = vcovHC)
R> summary(biofilm.first7a1)

         Simultaneous Tests for General Linear Hypotheses

Multiple Comparisons of Means: Tukey Contrasts
```

```
Fit: aov(formula = OD595nm ~ Treatment, data = first7a1)

Linear Hypotheses:
                    Estimate Std. Error t value Pr(>|t|)
7a1V1 - 7a1 == 0     0.15324    0.02915   5.257 0.000364 ***
7a1V2 - 7a1 == 0     1.10676    0.14703   7.527  < 1e-04 ***
7a1V2 - 7a1V1 == 0   0.95352    0.14780   6.452  < 1e-04 ***
---
Signif. codes:  0 '***' 0.001 '**' 0.01 '*' 0.05 '.' 0.1 ' ' 1
(Adjusted p values reported -- single-step method)
```

Apply **multcomp** statistics (Hothorn *et al.* 2008; Herberich *et al.* 2010) to the ***second*** replicate on biofilm analysis of strain 7a1 and its variants. The stars indicate the level of statistical significance between pairwise comparison of groups.

```
R> amod <- aov(OD595nm ~ Treatment, data = second7a1)
R> biofilm.second7a1 <- glht(amod, mcp(Treatment = "Tukey"), vcov = vcovHC)
R> summary(biofilm.second7a1)

         Simultaneous Tests for General Linear Hypotheses

Multiple Comparisons of Means: Tukey Contrasts


Fit: aov(formula = OD595nm ~ Treatment, data = second7a1)

Linear Hypotheses:
                    Estimate Std. Error t value Pr(>|t|)
7a1V1 - 7a1 == 0    -0.00178    0.01081  -0.165    0.985
7a1V2 - 7a1 == 0     0.54215    0.01409  38.480   <1e-05 ***
7a1V2 - 7a1V1 == 0   0.54393    0.01245  43.682   <1e-05 ***
---
Signif. codes:  0 '***' 0.001 '**' 0.01 '*' 0.05 '.' 0.1 ' ' 1
(Adjusted p values reported -- single-step method)
```

Apply **multcomp** statistics (Hothorn *et al.* 2008; Herberich *et al.* 2010) to the ***third*** replicate on biofilm analysis of strain 7a1 and its variants. The stars indicate the level of statistical significance between pairwise comparison of groups.

```
R> amod <- aov(OD595nm ~ Treatment, data = third7a1)
R> biofilm.third7a1 <- glht(amod, mcp(Treatment = "Tukey"), vcov = vcovHC)
R> summary(biofilm.third7a1)

         Simultaneous Tests for General Linear Hypotheses

Multiple Comparisons of Means: Tukey Contrasts
```

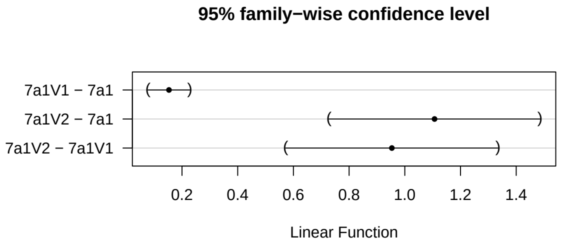

Figure 14: Plot of the statistical comparison of the first replicate on biofilm analysis of strain 7a1 and its variants. For interpretation of the plot, see legend of Figure 2.

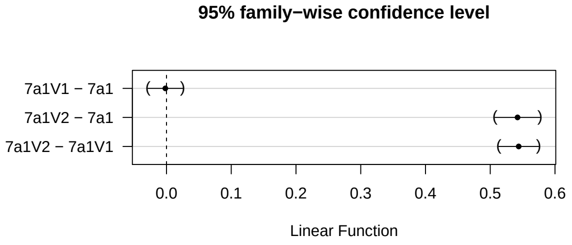

Figure 15: Plot of the statistical comparison of the second replicate on biofilm analysis of strain 7a1 and its variants. For interpretation of the plot, see legend of Figure 2.

```
Fit: aov(formula = OD595nm ~ Treatment, data = third7a1)

Linear Hypotheses:
                    Estimate Std. Error t value Pr(>|t|)
7a1V1 - 7a1 == 0     0.10124    0.02070   4.890   <1e-04 ***
7a1V2 - 7a1 == 0     0.33534    0.02026  16.554   <1e-04 ***
7a1V2 - 7a1V1 == 0   0.23410    0.02796   8.372   <1e-04 ***
---
Signif. codes:  0 '***' 0.001 '**' 0.01 '*' 0.05 '.' 0.1 ' ' 1
(Adjusted p values reported -- single-step method)
```

3.6. Statistics 7a1 biofilm: plot of significance tests

Below is the R code for Figure 14.

```
R> par(mai = c(1,2.0,1,0.5))
R> plot(biofilm.first7a1)
```

Below is the R code for Figure 15.

```
R> par(mai = c(1,2.0,1,0.5))
R> plot(biofilm.second7a1)
```

Below is the R code for Figure 16.

```
R> par(mai = c(1,2.0,1,0.5))
R> plot(biofilm.third7a1)
```

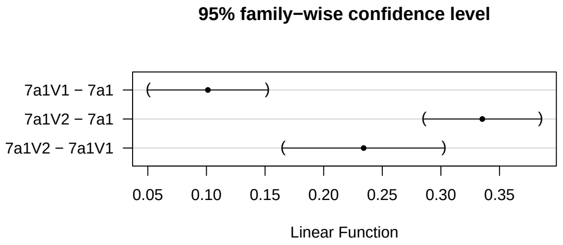

Figure 16: Plot of the statistical comparison of the third replicate on biofilm analysis of strain 7a1 and its variants. For interpretation of the plot, see legend of Figure 2.

4. Analysis of OmniLog GenIII microplate data

Details on the OmniLog® technology have been published elsewhere (Bochner, Gadzinski, and Panomitros 2001; Bochner 2009).

The raw data have been compiled to an R object using the funcionalities described in the vignette (the user-friendly hands-on manual) of the R package **opm** (Vaas, Sikorski, Michael, Göker, and Klenk 2012). All web resources regarding **opm** are linked on its main website `http://opm.dsmz.de/`. The data are loaded:

```
R> load("phenotypic variants.RData")
```

4.1. GenIII data strain M6

Below is the code in order to reproduce Figure 17.

```
R> xy_plot(subset(x, query = list(strain = "M6",
                                  experiment = c("first", "second","third")))
           [,,c("B11", "C03", "F09", "G03", "G06", "H03")],
           col = c("black", "black", "black","red", "red", "red",
                   "blue", "blue", "blue"),
               include  =  list("strain", "variant", "experiment"), lwd = 0.1,
               main = "Phenotypic variants of M6",
               neg.ctrl = 50,base.col = "darkred", base.lwd = 1,
               legend.fmt  = list(space = "right"),
               striptext.fmt = list(cex = 1))
```

Subset the data for only those curves displayed in Figure 17.

```
R> strainM6 <- opm::subset(x, query = list(strain = "M6",
                experiment = c("first", "second","third")))
                [,,c("B11", "C03", "F09", "G03", "G06", "H03")]
```

Extract the aggregated curve parameter values for the "Area under the curve"(AUC) as a data frame.

```
R> strainM6Table <- opm::extract(strainM6, dataframe = TRUE,
     as.labels = list("strain.variant"),  subset = "AUC")
```

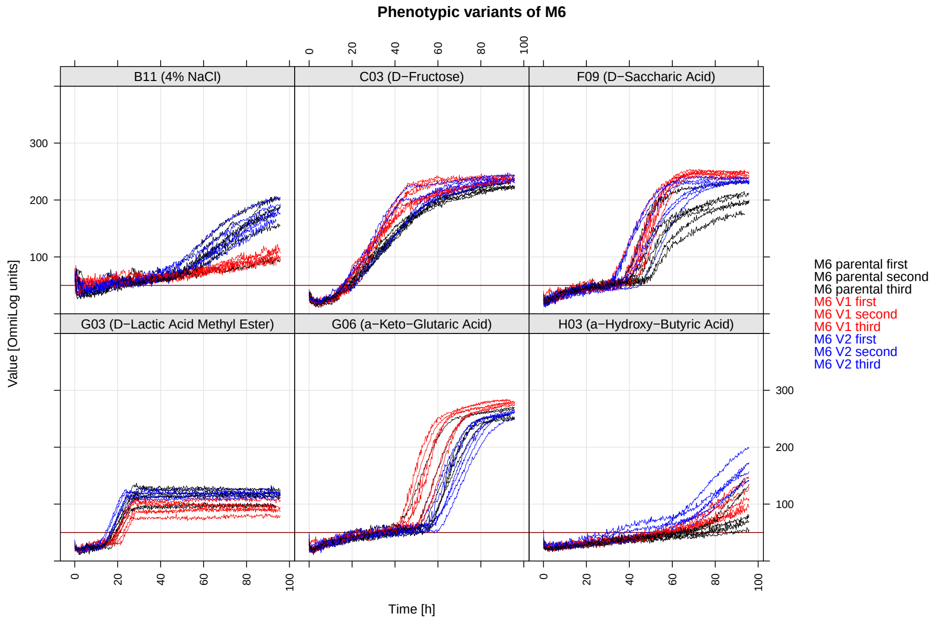

Figure 17: Dynamics of tetrazolium reduction in selected Biolog GEN III MicroPlate wells of strain M6. Six wells showing distinguished patterns between parental strains and variants are shown for M6.

4.2. multcomp statistics for strain M6, well B11 (4% NaCl)

Apply **multcomp** statistics (Hothorn *et al.* 2008; Herberich *et al.* 2010) to the AUC curve parameter values of strain M6 for well ***B11 (4% NaCl)***. The stars indicate the level of statistical significance between pairwise comparison of groups.

```
R> amod <- aov(strainM6Table[, "B11 (4% NaCl)"] ~ strain.variant,
                 data = strainM6Table)
R> B11M6 <- glht(amod, mcp(strain.variant = "Tukey"), vcov = vcovHC)
R> summary(B11M6)

         Simultaneous Tests for General Linear Hypotheses

Multiple Comparisons of Means: Tukey Contrasts


Fit: aov(formula = strainM6Table[, "B11 (4% NaCl)"] ~ strain.variant,
    data = strainM6Table)

Linear Hypotheses:
                         Estimate Std. Error t value Pr(>|t|)
M6_V1 - M6_parental == 0  -1726.6      549.4  -3.143   0.0166 *
M6_V2 - M6_parental == 0    451.6      665.3   0.679   0.7726
M6_V2 - M6_V1 == 0         2178.2      435.5   5.002   <0.001 ***
---
```

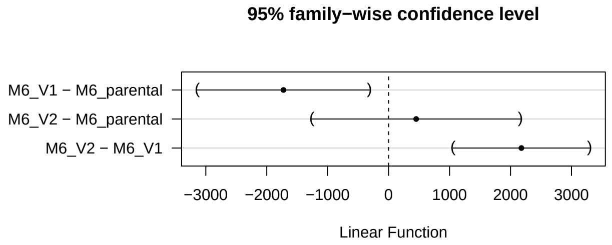

Figure 18: Plot of the statistical comparison of AUC value from the respiration curve kinetics of strain M6 in the well B11 (4% NaCl). For interpretation of the plot, see legend of Figure 2.

```
Signif. codes:  0 '***' 0.001 '**' 0.01 '*' 0.05 '.' 0.1 ' ' 1
(Adjusted p values reported -- single-step method)
```

Below is the R code for Figure 18.

```
R> par(mai = c(1,3,1,0.5))
R> plot(B11M6)
```

4.3. multcomp statistics for strain M6, well C03 (D-Fructose)

Apply **multcomp** statistics (Hothorn *et al.* 2008; Herberich *et al.* 2010) to the AUC curve parameter values of strain M6 for well ***C03 (D-Fructose)***. The stars indicate the level of statistical significance between pairwise comparison of groups.

```
R> amod <- aov(strainM6Table[, "C03 (D-Fructose)"] ~ strain.variant,
               data = strainM6Table)
R> C03M6 <- glht(amod, mcp(strain.variant = "Tukey"), vcov = vcovHC)
R> summary(C03M6)

         Simultaneous Tests for General Linear Hypotheses

Multiple Comparisons of Means: Tukey Contrasts


Fit: aov(formula = strainM6Table[, "C03 (D-Fructose)"] ~ strain.variant,
    data = strainM6Table)

Linear Hypotheses:
                         Estimate Std. Error t value Pr(>|t|)
M6_V1 - M6_parental == 0   1841.5      269.0   6.845   <0.001 ***
M6_V2 - M6_parental == 0   1050.6      523.5   2.007    0.137
M6_V2 - M6_V1 == 0         -790.9      547.7  -1.444    0.332
---
Signif. codes:  0 '***' 0.001 '**' 0.01 '*' 0.05 '.' 0.1 ' ' 1
(Adjusted p values reported -- single-step method)
```

Below is the R code for Figure 19.

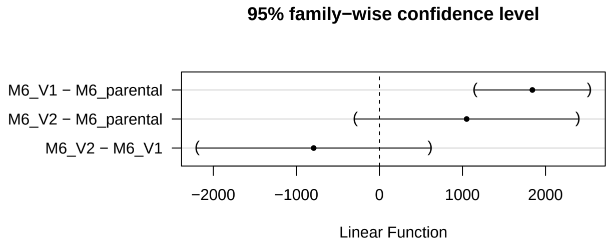

Figure 19: Plot of the statistical comparison of AUC value from the respiration curve kinetics of strain M6 in the well C03 (D-Fructose). For interpretation of the plot, see legend of Figure 2.

```
R> par(mai = c(1,3,1,0.5))
R> plot(C03M6)
```

4.4. multcomp statistics for strain M6, well F09 (D-Saccharic Acid)

Apply **multcomp** statistics (Hothorn *et al.* 2008; Herberich *et al.* 2010) to the AUC curve parameter values of strain M6 for well ***F09 (D-Saccharic Acid)*** . The stars indicate the level of statistical significance between pairwise comparison of groups.

```
R> amod <- aov(strainM6Table[, "F09 (D-Saccharic Acid)"] ~ strain.variant,
               data = strainM6Table)
R> F09M6 <- glht(amod, mcp(strain.variant = "Tukey"), vcov = vcovHC)
R> summary(F09M6)

         Simultaneous Tests for General Linear Hypotheses

Multiple Comparisons of Means: Tukey Contrasts


Fit: aov(formula = strainM6Table[, "F09 (D-Saccharic Acid)"] ~ strain.variant,
    data = strainM6Table)

Linear Hypotheses:
                         Estimate Std. Error t value Pr(>|t|)
M6_V1 - M6_parental == 0   2994.1      759.5   3.942  0.00344 **
M6_V2 - M6_parental == 0   2197.6      962.9   2.282  0.08661 .
M6_V2 - M6_V1 == 0         -796.5      696.3  -1.144  0.49481
---
Signif. codes:  0 '***' 0.001 '**' 0.01 '*' 0.05 '.' 0.1 ' ' 1
(Adjusted p values reported -- single-step method)
```

Below is the R code for Figure 20.

```
R> par(mai = c(1,3,1,0.5))
R> plot(F09M6)
```

4.5. multcomp statistics for strain M6, well G03 (D-Lactic Acid Methyl Ester)

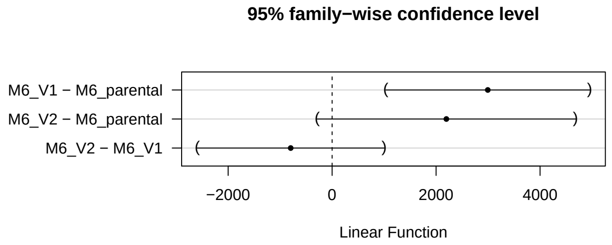

Figure 20: Plot of the statistical comparison of AUC value from the respiration curve kinetics of strain M6 in the well F09 (D-Saccharic Acid). For interpretation of the plot, see legend of Figure 2.

Apply **multcomp** statistics (Hothorn *et al.* 2008; Herberich *et al.* 2010) to the AUC curve parameter values of strain M6 for well ***G03 (D-Lactic Acid Methyl Ester)***. The stars indicate the level of statistical significance between pairwise comparison of groups.

```
R> amod <- aov(strainM6Table[, "G03 (D-Lactic Acid Methyl Ester)"] ~ strain.variant,
               data = strainM6Table)
R> G03M6 <- glht(amod, mcp(strain.variant = "Tukey"), vcov = vcovHC)
R> summary(G03M6)
```

```
         Simultaneous Tests for General Linear Hypotheses

Multiple Comparisons of Means: Tukey Contrasts


Fit: aov(formula = strainM6Table[, "G03 (D-Lactic Acid Methyl Ester)"] ~ 
    strain.variant, data = strainM6Table)

Linear Hypotheses:
                         Estimate Std. Error t value Pr(>|t|)    
M6_V1 - M6_parental == 0  -1434.0      612.6  -2.341   0.0778 .  
M6_V2 - M6_parental == 0    562.1      532.7   1.055   0.5476    
M6_V2 - M6_V1 == 0         1996.1      403.9   4.942   <0.001 ***
---
Signif. codes:  0 '***' 0.001 '**' 0.01 '*' 0.05 '.' 0.1 ' ' 1
(Adjusted p values reported -- single-step method)
```

Below is the R code for Figure 21.

```
R> par(mai = c(1,3,1,0.5))
R> plot(G03M6)
```

4.6. multcomp statistics for strain M6, well ”G06 (a-Keto-Glutaric Acid)”

Apply **multcomp** statistics (Hothorn *et al.* 2008; Herberich *et al.* 2010) to the AUC curve parameter values of strain M6 for well ***G06 (a-Keto-Glutaric Acid)***. The stars indicate the level of statistical significance between pairwise comparison of groups.

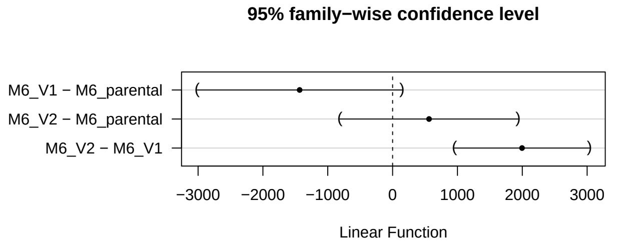

Figure 21: Plot of the statistical comparison of AUC value from the respiration curve kinetics of strain M6 in the well G03 (D-Lactic Acid Methyl Ester). For interpretation of the plot, see legend of Figure 2.

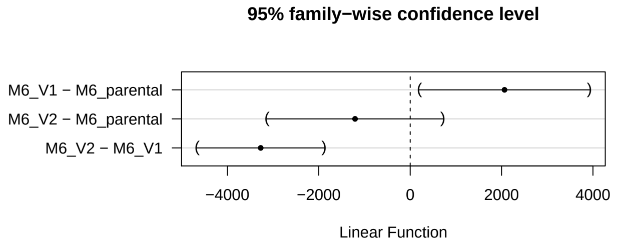

Figure 22: Plot of the statistical comparison of AUC value from the respiration curve kinetics of strain M6 in the well G06 (a-Keto-Glutaric Acid). For interpretation of the plot, see legend of Figure 2.

```
R> amod <- aov(strainM6Table[, "G06 (a-Keto-Glutaric Acid)"] ~ strain.variant,
               data = strainM6Table)
R> G06M6 <- glht(amod, mcp(strain.variant = "Tukey"), vcov = vcovHC)
R> summary(G06M6)

         Simultaneous Tests for General Linear Hypotheses

Multiple Comparisons of Means: Tukey Contrasts


Fit: aov(formula = strainM6Table[, "G06 (a-Keto-Glutaric Acid)"] ~
    strain.variant, data = strainM6Table)

Linear Hypotheses:
                         Estimate Std. Error t value Pr(>|t|)
M6_V1 - M6_parental == 0   2064.5      719.7   2.869   0.0291 *
M6_V2 - M6_parental == 0  -1206.6      743.8  -1.622   0.2636
M6_V2 - M6_V1 == 0        -3271.1      537.3  -6.088   <0.001 ***
---
Signif. codes:  0 '***' 0.001 '**' 0.01 '*' 0.05 '.' 0.1 ' ' 1
(Adjusted p values reported -- single-step method)
```

Below is the R code for Figure 22.

```
R> par(mai = c(1,3,1,0.5))
R> plot(G06M6)
```

4.7. multcomp statistics for strain M6, well H03 (a-Hydroxy-Butyric Acid)

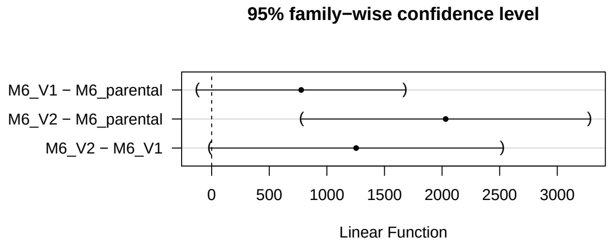

Figure 23: Plot of the statistical comparison of AUC value from the respiration curve kinetics of strain M6 in the well H03 (a-Hydroxy-Butyric Acid). For interpretation of the plot, see legend of Figure 2.

Apply **multcomp** statistics (Hothorn *et al.* 2008; Herberich *et al.* 2010) to the AUC curve parameter values of strain M6 for well ***H03 (a-Hydroxy-Butyric Acid)***. The stars indicate the level of statistical significance between pairwise comparison of groups.

```
R> amod <- aov(strainM6Table[, "H03 (a-Hydroxy-Butyric Acid)"] ~ strain.variant,
               data = strainM6Table)
R> H03M6 <- glht(amod, mcp(strain.variant = "Tukey"), vcov = vcovHC)
R> summary(H03M6)

         Simultaneous Tests for General Linear Hypotheses

Multiple Comparisons of Means: Tukey Contrasts


Fit: aov(formula = strainM6Table[, "H03 (a-Hydroxy-Butyric Acid)"] ~
    strain.variant, data = strainM6Table)

Linear Hypotheses:
                         Estimate Std. Error t value Pr(>|t|)
M6_V1 - M6_parental == 0    777.1      348.7   2.228  0.09683 .
M6_V2 - M6_parental == 0   2031.1      483.3   4.202  0.00201 **
M6_V2 - M6_V1 == 0         1254.1      490.9   2.555  0.05276 .
---
Signif. codes:  0 '***' 0.001 '**' 0.01 '*' 0.05 '.' 0.1 ' ' 1
(Adjusted p values reported -- single-step method)
```

Below is the R code for Figure 23.

```
R> par(mai = c(1,3,1,0.5))
R> plot(H03M6)
```

4.8. GenIII data strain 7a1

Below is the code in order to reproduce Figure 24.

```
R> xy_plot(subset(x, query = list(strain = "7a1",
                                  experiment = c("first", "second","third")))
```

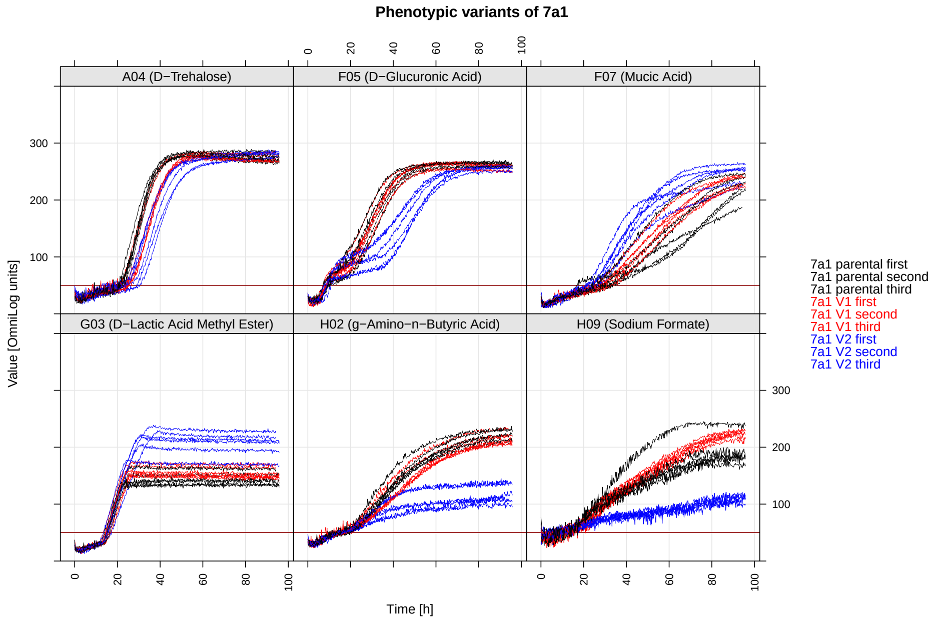

Figure 24: Dynamics of tetrazolium reduction in selected Biolog GEN III MicroPlate wells of strain 7a1. Six wells showing distinguished patterns between parental strains and variants are shown for 7a1.

```
[,,c("A04", "F05", "F07", "G03", "H02", "H09")],
col = c("black", "black", "black","red", "red", "red",
            "blue", "blue", "blue"),
    include  =  list("strain", "variant", "experiment"), lwd = 0.1,
    main = "Phenotypic variants of 7a1",
    neg.ctrl = 50,base.col = "darkred", base.lwd = 1,
    legend.fmt  = list(space = "right"),
    striptext.fmt = list(cex = 1))
```

Subset the data for only those curves displayed in Figure 24.

```
R> strain7a1 <- opm::subset(x, query = list(strain = "7a1",
                   experiment = c("first", "second","third")))
                   [,,c("A04", "F05", "F07", "G03", "H02", "H09")]
```

Extract the aggregated curve parameter values for the “Area under the curve”(AUC) as a data frame.

```
R> strain7a1Table <- opm::extract(strain7a1, dataframe = TRUE,
     as.labels = list("strain.variant"),  subset = "AUC")
```

4.9. multcomp statistics for strain 7a1, well A04 (D-Trehalose)

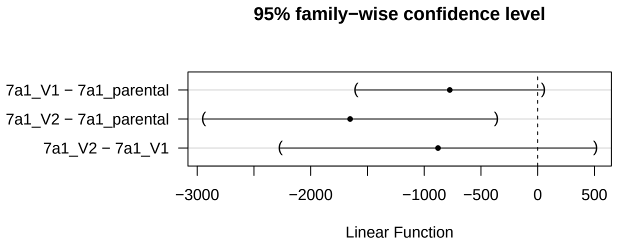

Figure 25: Plot of the statistical comparison of AUC value from the respiration curve kinetics of strain 7a1 in the well A04 (D-Trehalose). For interpretation of the plot, see legend of Figure 2.

Apply **multcomp** statistics (Hothorn *et al.* 2008; Herberich *et al.* 2010) to the AUC curve parameter values of strain 7a1 for well ***A04 (D-Trehalose)***. The stars indicate the level of statistical significance between pairwise comparison of groups.

```
R> amod <- aov(strain7a1Table[, "A04 (D-Trehalose)"] ~ strain.variant,
               data = strain7a1Table)
R> A047a1 <- glht(amod, mcp(strain.variant = "Tukey"), vcov = vcovHC)
R> summary(A047a1)

         Simultaneous Tests for General Linear Hypotheses

Multiple Comparisons of Means: Tukey Contrasts


Fit: aov(formula = strain7a1Table[, "A04 (D-Trehalose)"] ~ strain.variant,
    data = strain7a1Table)

Linear Hypotheses:
                           Estimate Std. Error t value Pr(>|t|)
7a1_V1 - 7a1_parental == 0   -774.6      320.9  -2.414   0.0676 .
7a1_V2 - 7a1_parental == 0  -1652.8      501.4  -3.296   0.0120 *
7a1_V2 - 7a1_V1 == 0         -878.2      539.7  -1.627   0.2576
---
Signif. codes:  0 '***' 0.001 '**' 0.01 '*' 0.05 '.' 0.1 ' ' 1
(Adjusted p values reported -- single-step method)
```

Below is the R code for Figure 25.

```
R> par(mai = c(1,3,1,0.5))
R> plot(A047a1)
```

4.10. multcomp statistics for strain 7a1, well F05 (D-Glucuronic Acid)

Apply **multcomp** statistics (Hothorn *et al.* 2008; Herberich *et al.* 2010) to the AUC curve parameter values of strain 7a1 for well ***F05 (D-Glucuronic Acid)***. The stars indicate the level of statistical significance between pairwise comparison of groups.

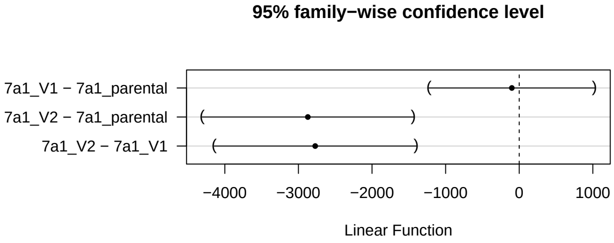

Figure 26: Plot of the statistical comparison of AUC value from the respiration curve kinetics of strain 7a1 in the well F05 (D-Glucuronic Acid). For interpretation of the plot, see legend of Figure 2.

```
R> amod <- aov(strain7a1Table[, "F05 (D-Glucuronic Acid)"] ~ strain.variant,
               data = strain7a1Table)
R> F057a1 <- glht(amod, mcp(strain.variant = "Tukey"), vcov = vcovHC)
R> summary(F057a1)

         Simultaneous Tests for General Linear Hypotheses

Multiple Comparisons of Means: Tukey Contrasts


Fit: aov(formula = strain7a1Table[, "F05 (D-Glucuronic Acid)"] ~ strain.variant,
    data = strain7a1Table)

Linear Hypotheses:
                           Estimate Std. Error t value Pr(>|t|)
7a1_V1 - 7a1_parental == 0   -100.4      433.8  -0.231    0.971
7a1_V2 - 7a1_parental == 0  -2872.3      553.7  -5.187   <0.001 ***
7a1_V2 - 7a1_V1 == 0        -2772.0      529.2  -5.238   <0.001 ***
---
Signif. codes:  0 '***' 0.001 '**' 0.01 '*' 0.05 '.' 0.1 ' ' 1
(Adjusted p values reported -- single-step method)
```

Below is the R code for Figure 26.

```
R> par(mai = c(1,3,1,0.5))
R> plot(F057a1)
```

4.11. multcomp statistics for strain 7a1, well F07 (Mucic Acid)

Apply **multcomp** statistics (Hothorn *et al.* 2008; Herberich *et al.* 2010) to the AUC curve parameter values of strain 7a1 for well ***F07 (Mucic Acid)***. The stars indicate the level of statistical significance between pairwise comparison of groups.

```
R> amod <- aov(strain7a1Table[, "F07 (Mucic Acid)"] ~ strain.variant,
               data = strain7a1Table)
R> F077a1 <- glht(amod, mcp(strain.variant = "Tukey"), vcov = vcovHC)
R> summary(F077a1)
```

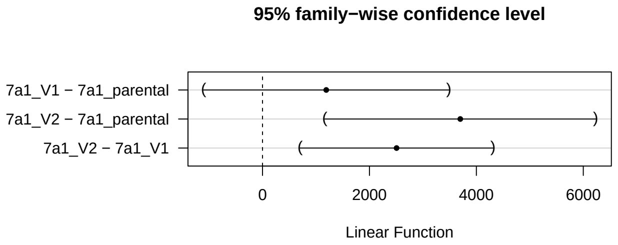

Figure 27: Plot of the statistical comparison of AUC value from the respiration curve kinetics of strain 7a1 in the well F07 (Mucic Acid). For interpretation of the plot, see legend of Figure 2.

```
         Simultaneous Tests for General Linear Hypotheses

Multiple Comparisons of Means: Tukey Contrasts


Fit: aov(formula = strain7a1Table[, "F07 (Mucic Acid)"] ~ strain.variant,
    data = strain7a1Table)

Linear Hypotheses:
                           Estimate Std. Error t value Pr(>|t|)
7a1_V1 - 7a1_parental == 0   1192.0      887.5   1.343  0.38959
7a1_V2 - 7a1_parental == 0   3699.4      980.0   3.775  0.00490 **
7a1_V2 - 7a1_V1 == 0         2507.4      698.2   3.591  0.00673 **
---
Signif. codes:  0 '***' 0.001 '**' 0.01 '*' 0.05 '.' 0.1 ' ' 1
(Adjusted p values reported -- single-step method)
```

Below is the R code for Figure 27.

```
R> par(mai = c(1,3,1,0.5))
R> plot(F077a1)
```

4.12. multcomp statistics for strain 7a1, well G03 (D-Lactic Acid Methyl Ester)

Apply **multcomp** statistics (Hothorn *et al.* 2008; Herberich *et al.* 2010) to the AUC curve parameter values of strain 7a1 for well ***G03 (D-Lactic Acid Methyl Ester)***. The stars indicate the level of statistical significance between pairwise comparison of groups.

```
R> amod <- aov(strain7a1Table[, "G03 (D-Lactic Acid Methyl Ester)"] ~ strain.variant,
               data = strain7a1Table)
R> G037a1 <- glht(amod, mcp(strain.variant = "Tukey"), vcov = vcovHC)
R> summary(G037a1)

         Simultaneous Tests for General Linear Hypotheses

Multiple Comparisons of Means: Tukey Contrasts
```

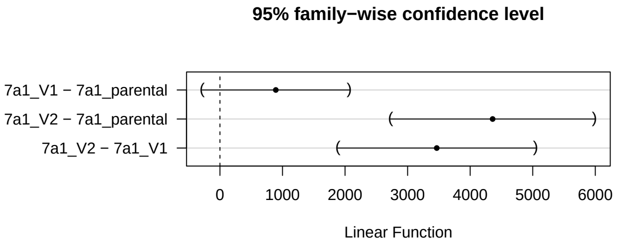

Figure 28: Plot of the statistical comparison of AUC value from the respiration curve kinetics of strain 7a1 in the well G03 (D-Lactic Acid Methyl Ester). For interpretation of the plot, see legend of Figure 2.

```
Fit: aov(formula = strain7a1Table[, "G03 (D-Lactic Acid Methyl Ester)"] ~
    strain.variant, data = strain7a1Table)

Linear Hypotheses:
                           Estimate Std. Error t value Pr(>|t|)
7a1_V1 - 7a1_parental == 0    892.9      454.5   1.964    0.153
7a1_V2 - 7a1_parental == 0   4358.9      629.8   6.921   <0.001 ***
7a1_V2 - 7a1_V1 == 0         3465.9      610.3   5.679   <0.001 ***
---
Signif. codes:  0 '***' 0.001 '**' 0.01 '*' 0.05 '.' 0.1 ' ' 1
(Adjusted p values reported -- single-step method)
```

Below is the R code for Figure 28.

```
R> par(mai = c(1,3,1,0.5))
R> plot(G037a1)
```

4.13. multcomp statistics for strain 7a1, well H02 (g-Amino-n-Butyric Acid)

Apply **multcomp** statistics (Hothorn *et al.* 2008; Herberich *et al.* 2010) to the AUC curve parameter values of strain 7a1 for well ***H02 (g-Amino-n-Butyric Acid)***. The stars indicate the level of statistical significance between pairwise comparison of groups.

```
R> amod <- aov(strain7a1Table[, "H02 (g-Amino-n-Butyric Acid)"] ~ strain.variant,
              data = strain7a1Table)
R> H027a1 <- glht(amod, mcp(strain.variant = "Tukey"), vcov = vcovHC)
R> summary(H027a1)

         Simultaneous Tests for General Linear Hypotheses

Multiple Comparisons of Means: Tukey Contrasts


Fit: aov(formula = strain7a1Table[, "H02 (g-Amino-n-Butyric Acid)"] ~
```

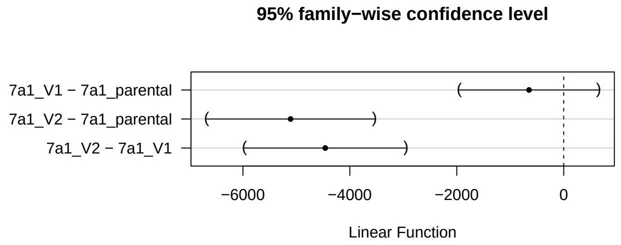

Figure 29: Plot of the statistical comparison of AUC value from the respiration curve kinetics of strain 7a1 in the well H02 (g-Amino-n-Butyric Acid). For interpretation of the plot, see legend of Figure 2.

```
    strain.variant, data = strain7a1Table)

Linear Hypotheses:
                           Estimate Std. Error t value Pr(>|t|)
7a1_V1 - 7a1_parental == 0   -648.3      502.2  -1.291     0.42
7a1_V2 - 7a1_parental == 0  -5108.1      605.5  -8.436   <0.001 ***
7a1_V2 - 7a1_V1 == 0        -4459.8      582.3  -7.658   <0.001 ***
---
Signif. codes:  0 '***' 0.001 '**' 0.01 '*' 0.05 '.' 0.1 ' ' 1
(Adjusted p values reported -- single-step method)
```

Below is the R code for Figure 29.

```
R> par(mai = c(1,3,1,0.5))
R> plot(H027a1)
```

4.14. multcomp statistics for strain 7a1, well H09 (Sodium Formate)

Apply **multcomp** statistics (Hothorn *et al.* 2008; Herberich *et al.* 2010) to the AUC curve parameter values of strain 7a1 for well ***H09 (Sodium Formate)***. The stars indicate the level of statistical significance between pairwise comparison of groups.

```
R> amod <- aov(strain7a1Table[, "H09 (Sodium Formate)"] ~ strain.variant,
               data = strain7a1Table)
R> H097a1 <- glht(amod, mcp(strain.variant = "Tukey"), vcov = vcovHC)
R> summary(H097a1)

         Simultaneous Tests for General Linear Hypotheses

Multiple Comparisons of Means: Tukey Contrasts


Fit: aov(formula = strain7a1Table[, "H09 (Sodium Formate)"] ~ strain.variant,
    data = strain7a1Table)

Linear Hypotheses:
                           Estimate Std. Error t value Pr(>|t|)
```

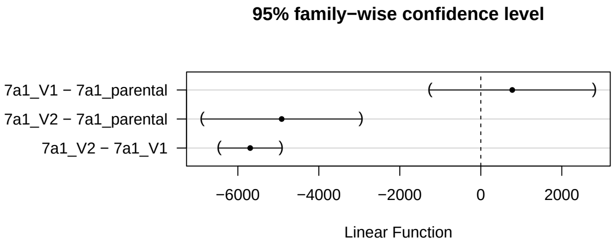

Figure 30: Plot of the statistical comparison of AUC value from the respiration curve kinetics of strain 7a1 in the well H09 (Sodium Formate). For interpretation of the plot, see legend of Figure 2.

```
7a1_V1 - 7a1_parental == 0    777.2      798.8   0.973    0.587
7a1_V2 - 7a1_parental == 0  -4918.1      771.2  -6.378   <1e-04 ***
7a1_V2 - 7a1_V1 == 0        -5695.3      301.1 -18.918   <1e-04 ***
---
Signif. codes:  0 '***' 0.001 '**' 0.01 '*' 0.05 '.' 0.1 ' ' 1
(Adjusted p values reported -- single-step method)
```

Below is the R code for Figure 30.

```
R> par(mai = c(1,3,1,0.5))
R> plot(H097a1)
```

References

Bochner B (2009). "Global Phenotypic Characterization of Bacteria." *FEMS Microbiological Reviews*, **33**, 191–205.

Bochner B, Gadzinski P, Panomitros E (2001). "Phenotype MicroArrays for High Throughput Phenotypic Testing and Assay of Gene Function." *Genome Research*, **11**, 1246–1255.

Herberich E, Sikorski J, Hothorn T (2010). "A Robust Procedure for Comparing Multiple Means under Heteroscedasticity in Unbalanced Designs." *PLoS ONE*, **5**(3), e9788.

Hothorn T, Bretz F, Westfall P (2008). "Simultaneous Inference in General Parametric Models." *Biometrical Journal*, **50**, 346–363. See vignette("generalsiminf", package = "multcomp").

Leisch F (2002). "Sweave: Dynamic Generation of Statistical Reports Using Literate Data Analysis." In W Härdle, B Rönz (eds.), *Compstat 2002 — Proceedings in Computational Statistics*, pp. 575–580. Physica Verlag, Heidelberg. ISBN 3-7908-1517-9, URL http://www.stat.uni-muenchen.de/~leisch/Sweave.

Vaas L, Sikorski J, Michael V, Göker M, Klenk H (2012). "Visualization and Curve Parameter Estimation Strategies for Efficient Exploration of Phenotype MicroArray Kinetics." *PLoS ONE*, **7**, e34846. See vignette("opm.pdf", package = "opm"), URL http://cran.r-project.org/web/packages/opm/vignettes/opm.pdf.

Affiliation:

Saul Burdmann
Department of Plant Pathology and Microbiology
The Robert H. Smith Faculty of Agriculture, Food and Environment
The Hebrew University of Jerusalem
P. O. Box 12, Rehovot 76100
Israel

Telephone: ++972-8-9489369
Fax: ++972-8-9466794
E-mail: saul.burdman@mail.huji.ac.il
URL: http://departments.agri.huji.ac.il/plantpath/burdman/
