# Supplementary figures and images for: Phenotypic Variation in the Plant Pathogenic Bacterium Acidovorax citrulli
Source: PLoS One. 2013 Sep 2;8(9):e73189. doi: 10.1371/journal.pone.0073189 (PMC3759439; doi:10.1371/journal.pone.0073189)

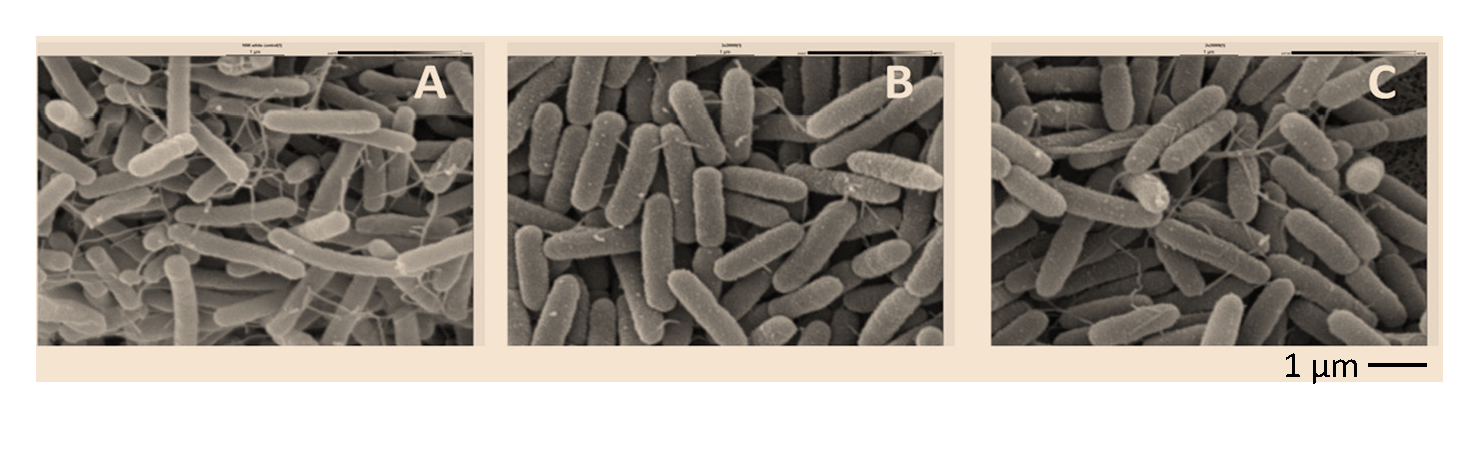

Supplement: Figure S1 — Scanning electron micrographs of cells from NA-grown colonies of A. citrulli M6 parental strain and variants. Fourty-eight-h-old colonies were excised from NA plates, fixed with glutaraldehyde, gradually dehydrated in ethanol, dried in a Critical Point Dryer, and finally coated with gold particles. Pictures were taken at a magnification level of 20,000X. A, parental strain M6; B, variant M6V1; C, variant M6V2. (TIFF) [file pone.0073189.s002.tiff]

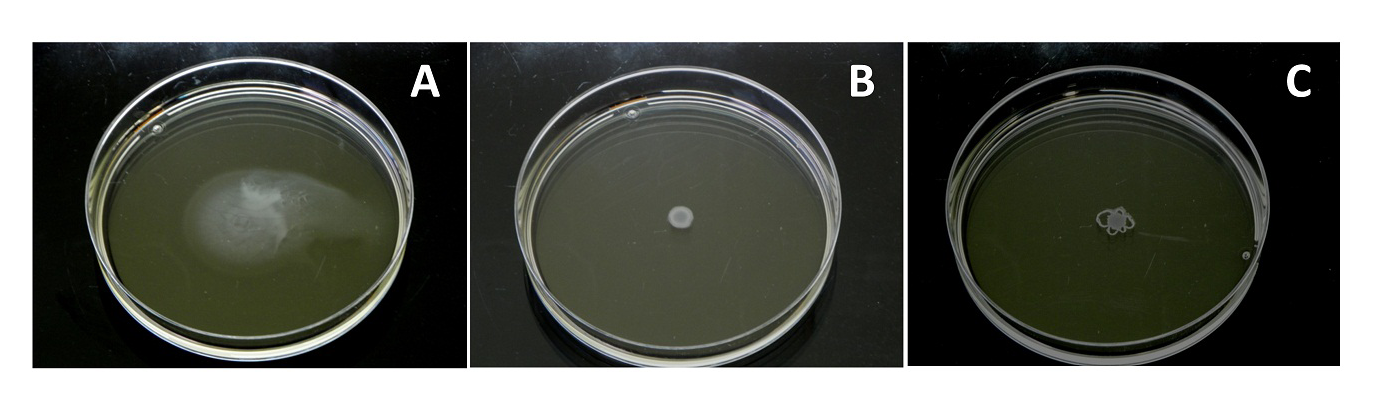

Supplement: Figure S2 — Images showing absence of swimming motility in PVs of A. citrulli 7a1. A, parental strain 7a1; B, variant 7a1V1; C, variant 7a1V2. Strains were inoculated onto the center of soft agar NA plates, and pictures were taken after 24 h of incubation at 28°C. Note the absence of swimming zones in the variants in contrast to the parental strain. (TIFF) [file pone.0073189.s003.tiff]

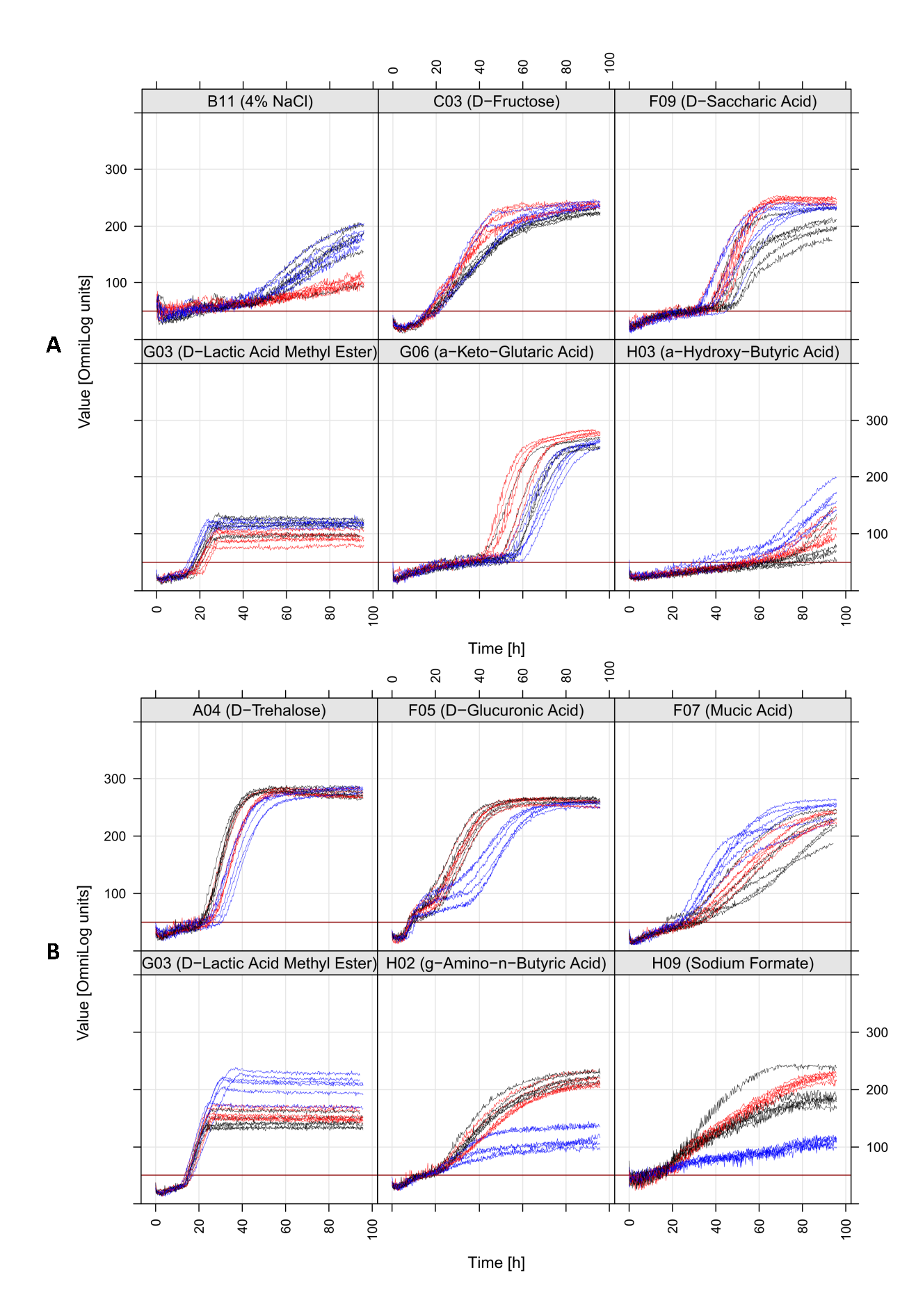

Supplement: Figure S3 — Dynamics of tetrazolium reduction in selected Biolog GEN III MicroPlate wells. Six wells showing distinguished patterns between parental strains and variants are shown for M6 (A) and 7a1 (B). For each strain, the parental strain (M6, 7a1) is colored black, variant 1 (M6V1, 7a1V1) is colored red, and variant 2 (M6V2, 7a1V2) is colored blue. Results are shown from three independent experiments, with two replicates per strain, yielding altogether six curves per strain. Details on statistical differences between parental strains and variants are shown in Appendix S1. (TIFF) [file pone.0073189.s004.tiff]
